# Structural and Functional Characterization of N-Glycanase-1 Pathogenic Variants

**DOI:** 10.3390/cells14131036

**Published:** 2025-07-07

**Authors:** Antje Banning, Lukas Hoeren, Isis Atallah, Ralph Orczyk, David Jacquier, Diana Ballhausen, Ritva Tikkanen

**Affiliations:** 1 Institute of Biochemistry, Medical Faculty, University of Giessen, Friedrichstrasse 24, DE-35390 Giessen, Germany; antje.banning@biochemie.med.uni-giessen.de (A.B.); lukas.hoeren@med.uni-giessen.de (L.H.); r.orczyk@gmx.de (R.O.); 2 Division of Genetic Medicine, Lausanne University Hospital and University of Lausanne, 1005 Lausanne, Switzerland; isis.atallah@unique-csmg.ch; 3 Pediatric Neurology and Neuro-Rehabilitation Unit, Woman-Mother-Child Department, Lausanne University Hospital and University of Lausanne, 1005 Lausanne, Switzerland; david.jacquier@chuv.ch; 4 Pediatric Metabolic Unit, Pediatrics, Woman-Mother-Child Department, Lausanne University Hospital and University of Lausanne, 1005 Lausanne, Switzerland; diana.ballhausen@chuv.ch

**Keywords:** congenital disorders of deglycosylation, N-Glycosylation, developmental delay, ERAD, protein misfolding, proteasome

## Abstract

NGLY1 deficiency is a congenital disorder of deglycosylation, caused by pathogenic variants of the *NGLY1* gene. It manifests as global developmental delay, hypo- or alacrima, hypotonia, and a primarily hyperkinetic movement disorder. The NGLY1 enzyme is involved in deglycosylation of misfolded N-glycosylated proteins before their proteasomal degradation and in the activation of transcription factors that control the expression of proteasomal subunits. Here, we have characterized the pathogenic NGLY1 variants found in three Swiss NGLY deficiency patients, as well as the most common pathogenic NGLY1 variant, Arg401*, found in about 20% of patients. Our functional and structural assessments of these variants show that they cause a profound reduction in NGLY1 activity, severely reduced expression of NGLY1 protein, and misprocessing of the transcription factor NFE2L1. Furthermore, transcription of proteasomal subunits and NGLY1 mRNA splicing are impaired by some of these variants. Our in silico structural analysis shows that the Arg390Gln substitution results in destabilization of NGLY1 structure due to a loss of an ionic interaction network of Arg390 and potentially impairment of protein–protein interactions. Our results provide important information on the functional and structural effects of pathogenic NGLY1 variants and pave the way for structure-based development of personalized treatment options.

## 1. Introduction

N-glycanase 1 (NGLY1, EC 3.5.1.52) is a cytosolic deglycosylating enzyme (peptide-N-glycanase or PNGase) that plays a critical role in the quality control of newly synthesized misfolded glycoproteins by removing N-glycans from Asn residues, thus facilitating an efficient degradation of the polypeptides in the proteasome [1,2]. In the absence of NGLY1 activity, N-linked carbohydrate chains can be partially removed from glycoproteins by the enzyme endo-β-N-acetylglucosaminidase (ENGase), leaving an Asn-linked N-acetyl glucosamine (GlcNAc) on the protein (reviewed in [3]).

**Figure 1:**
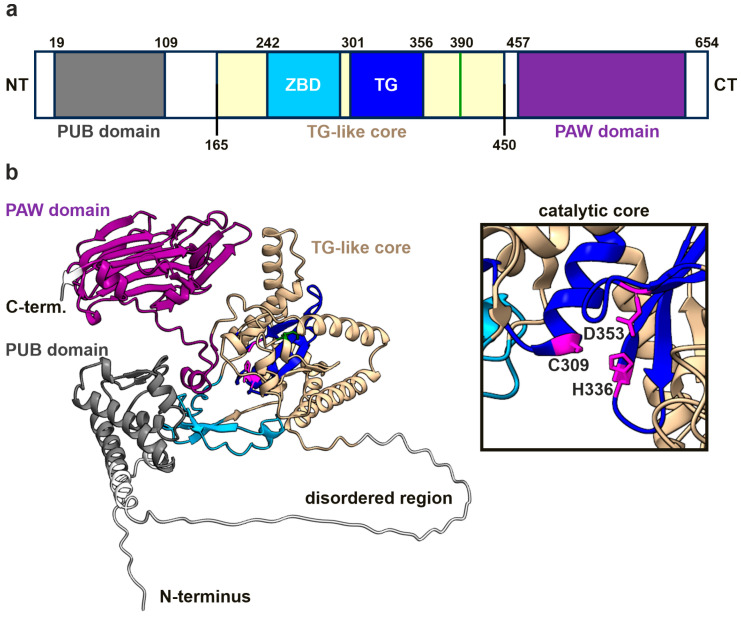
hNGLY1 protein structure predicted by AlphaFold3. (**a**) Domain structure of hNGLY1 containing the ubiquitin-binding domain (PUB; gray), the carbohydrate-binding domain (PAW; purple), and the transglutaminase-like core (beige) that contains the zinc-binding domain (ZBD; cyan) and the catalytic center (TG; blue). Domains adapted from [4] (*TG-like core*/ *ZBD*), [2] (*TG domain*), and [5] (*PUB* and *PAW*); green line indicates the position of R390. (**b**) The structure of hNGLY1 predicted by AlphaFold3, with the same color coding as in (**a**). The catalytic core of the TG domain as part of the TG-like core is shown in the magnified window, revealing the catalytic residues Cys309, His336, and Asp353 (in purple).

The NGLY1 enzyme contains several functional domains, including a PUB (*peptide:N-glycanase and UBA or UBX-containing proteins*) domain, a transglutaminase (TG)-like core region, and a PAW (*present in PNGases and other worm proteins*) domain (Figure 1a). The N-terminal PUB domain is a protein–protein interaction site connected with the TG-like domain by a flexible, disordered region, as illustrated in Figure 1b and Supplementary Figure S1 in an AlphaFold prediction of the human NGLY1 structure. The confidence of the prediction is generally high, except for the disordered region and the linker regions, which are more flexible (Supplementary Figure S1c). The root mean square deviation (RMSD) values for each domain (mNGLY1 vs. AlphaFold-predicted hNGLY1) are shown in Supplementary Table S1. The catalytic residues, Cys309, His336, and Asn353, reside within the TG-like core and form a catalytic triad for the hydrolysis of the N-glycosidic bond between the N-glycan chain and the Asn residue in the substrate. The C-terminal PAW domain in turn is responsible for the binding of the mannose residues within the carbohydrate chains of the substrate proteins.

NGLY1 deglycosylation activity is essential for the activation of the transcription factor NFE2L1 (Nuclear Factor Erythroid 2 Like 1, also referred to as Nrf1), which regulates the expression of proteasomal subunits [6,7,8]. NFE2L1 is synthesized as a single-span transmembrane protein that is partially translocated into the endoplasmic reticulum (ER) lumen, where it is N-glycosylated. Under normal cellular conditions, NFE2L1 is targeted for proteasomal degradation through the ER-associated degradation (ERAD) pathway. NGLY1 is essential for NFE2L1 activation in response to proteasome inhibition or under further cellular stress conditions. NFE2L1 activation requires its retrotranslocation, exposing the N-glycosylated part to deglycosylation by NGLY1 in the cytoplasm, combined with a proteolytic cleavage and subsequent release of NFE2L1 from the ER [8]. Thus, chemical or genetic ablation of NGLY1 activity results in an accumulation of misprocessed, inactive NFE2L1 within the cells [8]. Beyond the NFE2L1-mediated regulation of proteasomal subunits, NGLY1 has also been reported to act through NFE2L1 to influence mitochondrial function and inflammation [9,10].

NGLY1 deficiency (OMIM #615273) was described in 2012 as an inherited disease of deglycosylation (congenital disorder of deglycosylation 1 or CDDG1) [11]. It is an ultra-rare and severe multisystem disorder characterized by global developmental delay and/or intellectual disability, hypo- or alacrima, hypotonia, a primarily hyperkinetic movement disorder, and gait disturbances due to peripheral neuropathy [12,13,14,15]. Regression of milestones is frequently reported in NGLY1 deficiency, and the symptoms tend to be progressive. Biochemically, elevated liver transaminases are typical for NGLY1 deficiency, and the patients may exhibit excretion of aspartylglucosamine (GlcNAc-Asn) in the urine [16]. Over a half of the individuals with NGLY1 deficiency will develop a form of epilepsy (epileptic spasms and myoclonic and atonic seizures) in early childhood, with a mean age of onset at 43 months [14]. However, electroencephalogram abnormalities can be observed in 80% of all patients [14], and the median age of death is about 13 years [17]. Typical causes of death include respiratory failure and adrenal insufficiency, but cardiac arrest was also reported in a single case [17].

NGLY1 deficiency is caused by pathogenic variants in the *NGLY1* gene (OMIM* 610661; NM_018297.4) [15], and, to date, more than 130 pathogenic or likely pathogenic variants in *NGLY1* are included in the ClinVar [18] and GnomAD [19] databases. About half of the patients exhibit truncating nonsense variants that occur along the whole length of the protein, whereas frameshift mutations tend to be more concentrated in the C-terminal half of the protein [3]. Splice variants and in-frame deletions have also been reported in numerous patients. Missense mutations that result in a single amino acid change are present in less than 20% of the patients, and they tend to concentrate in the central part of the NGLY1 protein [3]. However, a detailed characterization of the structural and functional consequences of *NGLY1* missense variants at the protein level is frequently missing.

Here, we report three NGLY1 deficiency patients originating from two families and harboring three pathogenic/likely pathogenic variants. A previously uncharacterized *NGLY1* variant c.-17_12del; p? that results in a deletion of 29 bases surrounding the translation start site was identified, in combination with a previously described frame-shift variant, in one of the patients. Two siblings in the second family are homozygous for the missense variant c.1169G>A; p.Arg390Gln. Our functional studies confirmed the pathogenicity of the variants due to missing NGLY1 activity and/or expression and misprocessing of NFE2L1. We also show that the spectrum of NGLY1 mRNA isoforms is altered in the patient cells. Furthermore, we used in silico mutagenesis to predict the structural and thermodynamic consequences of the Arg390Gln exchange. Therefore, this study contributes to molecular characterization of the consequences of the pathogenic variants in NGLY1 deficiency and may pave the way to the development of personalized therapies based on understanding the structural and functional effects of individual patient variants.

## 2. Materials and Methods

### 2.1. Patient Description

#### 2.1.1. Patient 1

Patient 1 (P1) is a female who came to medical attention at the age of 24 months due to a developmental regression that was observed from the age of 17 months on. She is the first child of a non-consanguineous couple originating from Italy and Morocco, and she has a younger brother with normal psychomotor development. A maternal cousin, now an adult, was diagnosed with an autism spectrum disorder during childhood.

P1 was born from a spontaneous pregnancy with a standard ultrasound follow-up. The mother smoked cigarettes (half a package per day) throughout the pregnancy and occasionally consumed alcohol. Harmonious intrauterine growth retardation (IUGR) was discovered at 39 weeks of gestation, leading to an induced vaginal delivery. At birth, the child weighed 2140 g (<P3; <2.4 SD), with a length of 41 cm (<P3; 3.8 SD) and a head circumference of 31 cm (<P3; <2.6 SD). The child showed good neonatal adaptation.

During the first months of life, P1 showed catch-up growth, and her early motor development was within the normal range. At 17 months, she was able to stand and walk with support, started to eat alone with a spoon, and said a few words, but she was not able to point at objects. The parents reported a regression in language and motor development over the following months, impaired social interactions, and loss of interest in toys. There was no more eye contact during interactions with care persons. P1 became more isolated, selective with food, and irritable, with signs of auto-aggressive behavior. She also developed bruxism, but her sleep was of good quality. She had a tendency for constipation.

A comprehensive etiological assessment was performed, including brain magnetic resonance imaging with spectroscopy, electroencephalogram, and abdominal ultrasound, all of which were normal. The metabolic workup revealed mild but persistently increased values for transaminases, ASAT 56 U/l (normal values 9–45 U/l) and ALAT 83 U/l (8–38 U/l), that normalized beyond the age of 5 years. Urinary amino acids measured by Biochrome (DKSH International Ltd., Zurich, Switzerland) showed persistent, mildly elevated levels of GlcNAc-Asn (13–19 mmol/mol creatinine), suggesting a mild form of aspartylglucosaminuria (AGU).

Currently, P1 is 6 years old and goes to a specialized school for retarded children. Fatigue is a major problem in daily life. At the last examination, she showed normal growth for all three parameters and severe psychomotor retardation, with mild axial hypotonia. She walks independently and is followed by an ergotherapist to work on her fine motor skills. She does not establish eye contact and does not speak, so she only communicates non-verbally. A diagnosis of autism spectrum disorder has been established. She has started to sweat during physical exercise and now has tears when she cries. She is medicated for chronic constipation.

#### 2.1.2. Patient 2

Patient P2 is a female who is the third live-born child from the fourth pregnancy of first-degree cousins of Turkish origin. The first pregnancy of this couple ended with an intrauterine fetal demise at 38 weeks, but no investigation on the cause was performed. The first live-born child died from acute myeloblastic leukemia during childhood. The two older siblings of P2 showed normal psychomotor development. In the third term of pregnancy with P2, oligohydramnios was noted. The girl was born at term after provocation, with good neonatal adaptation (APGAR score 9/10/10).

At the age of 4 months, a vertical gaze palsy without other ophthalmological issues was noted, along with mild ataxia and hypomimia. She then presented a global development delay, with independent walking achieved at 30 months, and she spoke a few isolated words. An intermittent ptosis, often worsening towards the end of the day, became evident during the second year of life. She also suffered from substantial fatigability along with oromotor difficulties affecting both her speech and her swallowing abilities, leading to occasional aspirations. She also developed choreic movements, fluctuant myoclonic jerks, and sometimes a mild action tremor. All these motor manifestations worsened during febrile illnesses.

Several electroneurographies were performed throughout the years, some showing abnormal decrement on 3 Hz repetitive nerve stimulation. Medication trials with pyridostigmine, ephedrine, and salbutamol were performed for a potential myasthenic syndrome, but were stopped after a few weeks due to lack of efficiency (according to the family), or side effects, such as tremor. Electroencephalography did not show an irritative element, and brain magnetic resonance imaging was normal at 11 months and at 3 years and 7 months.

A mild but persistent elevation of transaminases was noted at the age of 22 months (ASAT 68 U/l, ALAT 43 U/l). Serum creatin kinase levels were mildly elevated between 7 and 14 months of age (233–292 U/l), with concurrent increases in transaminases (ASAT up to 125 U/l, ALAT up to 213 U/l, GGT 92 U/l), which progressively normalized by 3 years and 10 months. At the age of 16 years, transaminases were normal, and urinary amino acids measured by Biochrome showed only mildly elevated levels of GlcNAc-Asn (6 mmol/mol creatinine).

The patient is now 17 years old, has a mild-to-moderate intellectual disability, and attends a special needs facility. She walks independently but needs help with daily activities. The aforementioned motor elements (choreic movements, fluctuating ptosis, and swallowing difficulties) are still present.

#### 2.1.3. Patient 3

Patient P3 is the younger sister of P2, and she is the fourth child in the family. Her disease course resembles the description of her older sister, P2. During her first year of life, the neurological examination showed a hypomimia, fluctuating ptosis, occasional choreic movements, and a global hypotonia, but she had full eye movements. At the age of 2 years, a mixed polyneuropathy was diagnosed. At 23 months, ASAT was mildly elevated at 92 U/l and ALAT at 102 U/l, whereas at the age of 5 years and 7 months, ASAT was slightly above the normal limit at 50 U/l, but ALAT was normal. Assessment of urinary amino acids by Biochrome showed mildly elevated levels of GlcNAc-Asn (13 mmol/mol creatinine).

P3 is currently 7 years old and has a global developmental delay. She is ambulatory, with seemingly normal strength, axial hypotonia, progressive choreic movements, anterior drooling, and swallowing difficulties with occasional aspirations. All of these features worsen during febrile illnesses. She does not have tears when she cries, and she has bilateral fluctuating ptosis, together with a slightly limited vertical gaze. She can talk in short sentences but is not always intelligible. Fatigue is a considerable problem in daily life. She attends a special needs school, and she needs help with daily living activities.

### 2.2. Whole-Genome Sequencing

Peripheral blood samples from the patients and their parents were collected after the parents signed the informed consent form. Genomic DNA was extracted from peripheral blood leukocytes according to standard procedures. Whole-exome sequencing was conducted for P1 and P2 on a NextSeq 500 sequencer from Illumina using the Comprehensible library from Twist Biosciences^®^ (San Francisco, CA, USA) on genomic DNA extracted from leukocytes. The raw reads were aligned to the human reference genome GRCh37/hg19 with Novoalign (version 4.02.02, Novocraft Technologies; Selangor, Malaysia), and bioinformatics analysis was carried out using an in-house pipeline. Variant calling followed the Genome Analysis Toolkit best practice recommendations [20]. Copy number variation analysis was based on the ExomeDepth tool [21]. The analysis targeted 1,971 genes related to neurodevelopmental disorders (Supplementary Table S2). Familial segregation analysis was performed by Sanger sequencing.

### 2.3. Plasmids

The coding regions of human *NGLY1* and *NFE2L1* were PCR-amplified from HEK293T cDNA and cloned into pcDNA5/FRT or pcDNA3 vectors using BamHI/XhoI and HindIII/EcoRI digestions, respectively. The NGLY1 construct encoding isoform 1 served as a template for site-directed mutagenesis of Arg390 into Gln (R390Q) and of Arg401 into a stop codon (R401*). For identification of *NGLY1* isoforms, total RNA was isolated from patient and control cells, and the NGLY1 sequence was PCR-amplified using primers that would detect all possible isoforms. For this, primers that target the 5′ UTR upstream of the ATG start codon and 3′ UTR downstream of the stop codon were used. For patient P1, who is lacking the region around the ATG start codon, a different forward primer further upstream in the 5′ UTR was used (Table 1). All resulting PCR products representing different NGLY1 isoforms were cloned into a pcDNA3 vector. Several clones (12 each for control fibroblasts and patient P1; 7 each for patients P2 and P3) were sequenced, and the abundance of the isoforms was expressed as the percentage of the sequenced clones.

For reporter gene assays, the HO-ARE-pGL3prom construct, containing the antioxidant response element of heme oxygenase 1 [22], was used, together with a pRL-TK renilla luciferase vector (Promega, Mannheim, Germany) for normalization. For NGLY1 activity measurements, the deglycosylation-dependent ddVenus and deglycosylation-independent Venus constructs, pRetro-IRES-mCherry C3-SS-C-ddVenus, and pRetro-IRES-mCherry C3-SS-C-Venus [23], were kindly provided by Lars Steinmetz. These constructs served as templates for subcloning of Venus, ddVenus, and mCherry into a pcDNA3 vector. Venus and ddVenus subcloning was performed by PCR amplification with the primers EcoRI-SS-C-venus-fwd and Venus-NotI-stop rev, and mCherry was subcloned using mCherry-EcoRI-fwd and NotI-stop-mCherry-rev (Table 1). The correctness of all constructs was verified by sequencing (Microsynth Seqlab, Göttingen, Germany).

### 2.4. Cell Culture

Flp-In™-293 cells (Thermo Fisher Scientific, Dreieich, Germany) were cultured at 37 °C, 5% CO_2,_ in Dulbecco’s modified Eagle’s medium (DMEM) with high glucose, 10% fetal bovine serum (FBS), and 1% penicillin/streptomycin (all from Thermo Fisher Scientific). *NGLY1* knockout cells were produced using the CRISPR/Cas9 technique. The guide RNAs (gRNAs) were designed with the E-Crisp design tool (DKFZ, Heidelberg, Germany) and cloned using BbsI digestion into pSpCas9(BB)-2A-Puro (PX459, Addgene plasmid #48139). The procedure was identical to our previously published method [24]. NGLY1 knockout was confirmed by sequencing of the respective genomic region. For this purpose, genomic DNA was purified and PCR-amplified using the Phire Tissue Direct PCR Master Kit (Thermo Fisher Scientific), and NGLY1 mRNA level was analyzed by qPCR. All primer sequences are shown in Table 1.

Lack of protein expression was further confirmed by Western blotting. Stable cell lines overexpressing NGLY1 variants were generated by Flp recombinase-mediated integration, according to the Flp-In system manual (Thermo Fisher Scientific). In short, Flp-In^TM^-293 cells harbor an FRT recombination site downstream of a lacZ-zeocin fusion gene. For integration into this site, the *NGLY1* variants were cloned into pcDNA5/FRT, which, upon cotransfection with the Flp-recombinase (encoded by the pOG44 plasmid), recombines at the FRT site and creates hygromycin-resistant, but zeocin-sensitive, cells. Hygromycin-resistant clones were screened for expression of the target gene by Western blot. Correct recombination was confirmed by the lack of β-galactosidase activity by mixing 50 μL cell lysate with 70 μL β-galactosidase buffer (120 mM Na_2_HPO_4_, 80 mM NaH_2_PO_4_, 20 mM KCl, 2 mM MgCl_2_, 100 mM β-mercaptoethanol, pH 7.3) and with 30 μL ONPG 4 mg/mL solution in 60 mM Na_2_HPO_4_, pH 7.5 (Sigma-Aldrich, Taufkirchen, Germany). Samples were incubated at 37 °C until a clear yellow color was visible in the parental Flp-In™-293 cells. For stable overexpression of Venus and ddVenus, *NGLY1* knockout cells were stably transfected with ddVenus or Venus and selected with G418 (400 µg/mL).

Primary skin fibroblasts from the three NGLY1 deficiency patients were isolated from skin punch biopsies. Parents provided a signed informed consent form for the biopsy, fibroblast culture, storage, and enzymatic and molecular analyses (approval of the ethics committee of University of Giessen, #144/21). Then, 3–4 mm biopsies were stored in DMEM and shipped at room temperature. For the cultivation of primary fibroblasts, the biopsies were cut into at least 12 pieces that were distributed equally onto three 6 cm dishes containing 4 mL of AmnioMAX™ C-100 medium (Thermo Fisher Scientific). The dishes were kept in the incubator without interference, and, if necessary, the medium was replenished to avoid drying of the tissue. Fibroblasts were visible after two weeks. Confluent dishes were trypsinized and the cells transferred to several 10 cm dishes containing DMEM (high glucose), 10% FBS, 1% penicillin/streptomycin, 1% non-essential amino acids, and 1% sodium pyruvate. The cells were frozen as soon as possible. As controls, skin fibroblasts from different healthy donors were used, i.e., neonatal and adult fibroblasts from Innoprot (Derio, Spain), apparently healthy fibroblasts GM00969 (Coriell Institute of Medical Research, Camden, NJ, USA), and immortalized fibroblasts [25]. All cells were grown at 5% CO_2_ and 37 °C.

### 2.5. Enzyme Activity Measurements

The activity of aspartylglucosaminidase (AGA) in human serum and in cell lysates was measured as described in [26]. For NGLY1 activity measurements, Flp-In™-293 cells (parental, *NGLY1* knockout, or NGLY1-overexpressing cells), seeded onto 12-well plates, were transiently transfected with Venus and mCherry, or ddVenus and mCherry plasmids (250 ng each), respectively. After 24 h, the cells were transferred onto 6-well plates and incubated overnight with 1 µM of the proteasome inhibitor MG132 (Cayman Chemical, Biomol, Hamburg, Germany). Cells were lysed in 200 µL of lysis juice (PJK, Kleinblittersdorf, Germany), frozen overnight, sonicated for 3 s at 90% amplitude, and then centrifuged at 12,000× *g*, 3 min, 4 °C. An amount of 40 µL of cell lysate or lysis buffer (blank) was diluted in 100 µL of water, and the fluorescence of mCherry (ex 561 nm/em 610 nm) and Venus (ex 488 nm/em 530 nm) were measured with a Tecan infinite M200 plate reader (Tecan, Männedorf, Switzerland). Venus fluorescence was normalized to mCherry fluorescence.

To establish an NGLY1 activity assay that does not require transfection of cells, ddVenus-overexpressing *NGLY1* knockout cells were grown to confluency in 10 cm plates, treated with MG132 overnight, and then homogenized in 1 mL of Venus buffer (50 mM Tris-HCl pH 7.5, 10 mM sucrose, 5 mM EDTA, 1 mM DTT). Cell homogenates were sonicated on ice for 30 s, and 40 µL of this ddVenus-containing homogenate was mixed with 40 µL of sample (i.e., cell homogenate of HEK293 cells or fibroblasts in Venus buffer) and 100 µL of Venus buffer. The samples were incubated at room temperature, and Venus fluorescence was followed over time. For calculation, the background fluorescence of the blank sample (i.e., sample only containing ddVenus homogenate but no sample) was subtracted from all time points.

### 2.6. Reporter Gene Assays

For transient transfection, cells were seeded onto 12-well plates on the day before transfection. For transfections, 200 ng of reporter gene plasmid, 200 ng of NFE2L1 expression plasmid, and 50 ng of pRL-TK Renilla luciferase control reporter vector were transfected using MACSfectin™ (Miltenyi Biotec, Bergisch Gladbach, Germany) according to the manufacturer’s protocol. The following day, the cells were transferred onto 24-well plates and harvested after 24 h in lysis juice for a firefly and renilla luciferase assay (PJK). Determination of firefly and renilla luciferase activity was performed with a Tecan infinite M200 plate reader using 20 µL of lysate and 85 µL of beetle or renilla juice (both from PJK) as reagents. Relative luciferase activity was calculated by dividing firefly luciferase activity by renilla luciferase activity.

### 2.7. Western Blot

For analysis of total cell lysates, cells grown on 10 cm dishes were lysed in 50 mM Tris, pH 7.4, 150 mM NaCl, 2 mM EDTA, 1% Nonidet P-40, supplemented with Protease Inhibitor Mixture. For analysis of nuclear extracts, cells were gently resuspended in 1 mL lysis buffer (10 mM HEPES, pH 7.9, 1.5 mM MgCl_2_, 10 mM KCl, 0.5 mM DTT, 0.5 mM PMSF, 0.5% *v*/*v* Nonidet-P40) and lysed for 10 min at 4 °C. After centrifugation for 1 min, 7100× *g*, 4 °C, the supernatant (i.e., cytosolic fraction) was removed, and the resulting pellet was washed once in lysis buffer and then resuspended in 100 µL of nuclear extract buffer (40 mM HEPES, pH 7.9, 400 mM KCl, 10% (*v*/*v*) glycerol, 1 mM DTT, 0.1 mM PMSF). After addition of 6.25 µL of 5 M NaCl, the samples were lysed for 30 min on ice and finally centrifuged for 20 min, 20,800× *g*, 4 °C, to obtain a nuclear lysate. Equal protein amounts were analyzed with 10% SDS–polyacrylamide gel electrophoresis and Western blot on a nitrocellulose membrane. Primary antibodies were as follows: rabbit anti-NGLY1 (1:1000, either from Thermo Fisher scientific #PA5-110030 or from Sigma-Aldrich #HPA036825), rabbit anti-TCF11/NRF1 (1:1000, Cell Signaling, Leiden, Netherlands, #8052), rabbit anti-PARP1 (1:1000, Cell Signaling, #9532), and mouse anti-GAPDH (1:10,000, Abcam, Cambridge, UK; #ab-8245). Detection was performed with an Odyssey^®^ XF Imaging System (LI-COR Biotechnology, Bad Homburg, Germany).

### 2.8. RNA Isolation and Quantitative Real-Time PCR

For quantitative PCR (qPCR), total RNA was isolated with peqGOLD TriFast™ (VWR, Hannover, Germany), and a DNAse digestion was included. Total RNA (1–3 μg) was reverse-transcribed with 150 fmol oligo(dT) primers and M-MuLV reverse transcriptase (NEB, Frankfurt am Main, Germany) in a total volume of 45 μL. Real-time qPCRs were performed using the CFX Connect Real-Time PCR Detection System (Bio-Rad, Munich, Germany). The annealing temperature was 60 °C for all primers whose sequences are shown in Table 1. The reactions were carried out as duplicates with 20 ng cDNA in a total volume of 10 μL using iTaqTM Universal SYBR Green Supermix (Bio-Rad). PCR products were quantified with the ΔCt method. For normalization, the mean of the reference genes *B2M*, *Rpl13a*, *Ywhaz*, and *GAPDH* was used.

### 2.9. In Silico Mutagenesis and Analysis of Protein Stability

As no experimentally resolved structure of hNGLY1 is available, the X-ray crystal structure of the transglutaminase-like core domain of mNGLY1 (PDB ID: 2F4M; [4]) was subjected to in silico mutagenesis (Arg387Gln; equivalent to Arg390Gln in human NGLY1) using the DynaMut web tool [27]. Predictions of changes in protein stability (ΔΔG) and vibrational entropy (ΔΔSVib) were obtained through structure-based methods (SDM, DUET, and mCSM) and an NMA-based approach (ENCoM). The MASTROweb tool [28] was additionally employed to assess the mutation effects. Protein structures were visualized using UCSF ChimeraX software, version 1.9 [29].

To ensure sequence similarity between human (hNGLY1) and mouse (mNGLY1) TG-like core domains, protein sequences (hNGLY1: UniProt ID Q96IV0; mNGLY1: UniProt ID Q9JI78) were aligned using Serial Cloner, version 2.6.1 [30]. For structural comparison, hNGLY1 structure was predicted using AlphaFold 3 [31] and superimposed with the mNGLY1 TG-like core domain (PDB-ID: 2F4M). The root mean squared deviation (RMSD) of the Cα-atoms were calculated using ChimeraX and visualized using a color code (0.5 Å = blue; 1 Å = white; 1.5 Å = red). Domain annotations for NGLY1 were adapted from [4] (TG-like core, ZBD), [2] (TG domain), and [5] (PUB and PAW).

### 2.10. Statistical Analysis

All experiments were performed at least three times. The data are expressed as mean ± SD. Statistical comparisons between groups were made using Student’s *t*-tests or 1-way or 2-way ANOVA (Analysis of Variance), as appropriate, using GraphPad Prism 5 software (San Diego, CA, USA). Values of *p* < 0.05 were considered significant (*), while values of *p* < 0.01 were considered very significant (**) and *p* < 0.001 extremely significant (***).

## 3. Results

### 3.1. Pathogenic NGLY1 Variants in Two Families with NGLY1 Deficiency

**Figure 2:**
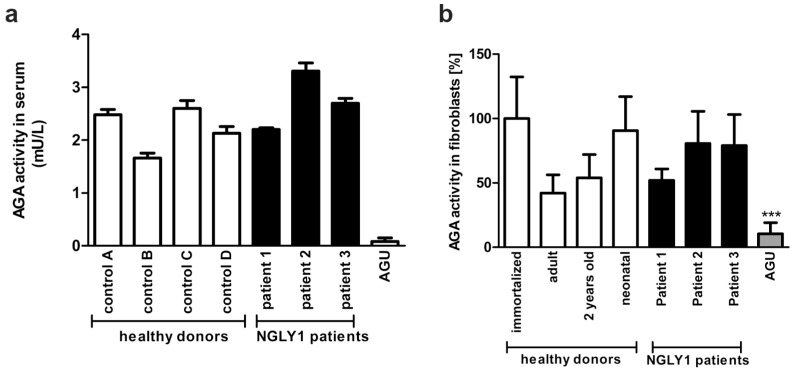
AGA enzyme activity in NGLY1 patients. (**a**) AGA activity was measured using equal amounts of serum of healthy controls and NGLY1 patients (n = 3). The mean of AGU patients (n = 20) was used as a control. (**b**) Fibroblasts from three NGLY1 patients, four healthy donors, and four AGU patients were grown on 10 cm dishes. AGA activity was measured in the cell lysates and normalized to total protein amount. Serum samples were measured in triplicate, and AGA activity in fibroblasts was measured 3 times for patients P2 and P3 and 6 times for patient P1 and the healthy donors. *** *p* < 0.001 AGU vs. all other samples.

The female patient 1 (P1) presented with a history of regression and with unspecific psychomotor developmental delay at the University Hospital of Lausanne, Switzerland. A detailed description of the patient is given in Section 2.1.1. A metabolic workup at 24 months revealed increased transaminase activity and elevated levels of GlcNAc-Asn in the urine, suggestive of aspartylglucosaminuria (AGU). However, the aspartylglucosaminidase (AGA) enzyme activity was within the normal range in the serum and in the fibroblasts (Figure 2a,b), ruling out AGU as a diagnosis.

Whole-exome sequencing (WES) was performed for clarification of a potential genetic origin of the developmental delay in P1. The WES initially focused on the analysis of 37 genes involved in Rett/Rett-like syndrome (characterized by episodes of developmental regression) and the *AGA* gene involved in AGU, but no pathogenic or likely pathogenic variants were discovered. The genetic analysis was then extended to 1971 genes involved in developmental disorders (listed in Supplementary Table S2).

Since NGLY1 deficiency can also cause high levels of urine GlcNAc-Asn in the absence of pathogenic *AGA* gene variants, a targeted analysis of the *NGLY1* gene was performed in P1 and her parents, revealing two compound heterozygous variants in the *NGLY1* gene: a paternal frameshift variant c.1370dupG, p.Arg458Lysfs* and a maternally inherited variant c.-17_12del; p? that results in a deletion of 29 base pairs from the 5′ untranslated region and exon 1, causing a loss of the ATG translation start codon. This variant is present in GnomAD, v.4.1.0 [19] with an allele frequency of 0.001492%, found mainly in the non-Finnish European population, and it is included in the ClinVar database [18] (VCV2637937), whereas the p.Arg458Lysfs14 variant is absent from the general population database (GnomAD; v.4.1.0) but listed in the ClinVar (VCV000126422) database. It was already described in a homozygous manner in a 20 year-old female patient originating from a consanguineous Italian family, with the clinical criteria for NGLY1 deficiency [13,15]. Thus, the diagnosis of NGLY1 deficiency was plausible for P1, even though she presented only with developmental delay and mild hypertransaminasemia, without the characteristic core features of NGLY1 deficiency, such as abnormal tear production, hyperkinetic movement disorder, or ataxia.

Two further female patients (P2 and P3, siblings of Turkish origin) with a similar phenotype were also diagnosed in the same university hospital as P1. WES revealed a homozygous missense variant c.1169G>A; p.Arg390Gln in the *NGLY1* gene, with both parents being heterozygous for this variant. The p.Arg390Gln variant is reported in GnomAD v4.1.0 with an allele frequency of 0.0000684%, and it was predicted to be deleterious by all variant predictors (CADD: 30.0, REVEL: 0.589, phyloP: 8.90, PolyPhen (max): 0.999). According to the American College of Medical Genetics and Genomics criteria [32], this variant is considered as “likely pathogenic”. It has also been reported in ClinVar (ID 827607) by an Iranian genetics center. Taken together, the presence of the likely pathogenic *NGLY1* variants in compound heterozygous or homozygous form, together with the observed disease symptoms and lack of evidence for AGU, confirmed the diagnosis of NGLY1 deficiency in all three patients.

### 3.2. NGLY1 Isoforms in Patient Fibroblasts

The *NGLY1* primary transcript is subject to alternative splicing [5], resulting in at least 13 isoforms of the human *NGLY1* mRNA [33], isoform number 1 being the longest and the best-characterized one. All other isoforms are a result of either exon skipping or the use of alternative exons, and the function of these isoforms is not known.

To investigate whether the patient variants lead to an altered *NGLY1* RNA expression or splicing, total RNA was isolated from dermal fibroblast cultures of two healthy individuals and the three patients and reversely transcribed, and the coding sequence of *NGLY1* was amplified by PCR, using primers that bind to the 5′ and 3′ UTR regions. The resulting products were separated on agarose gels and cloned into the pcDNA3 vector. Several clones (12 each for control fibroblasts and patient P1; 7 each for patients P2 and P3) were sequenced to gain insight into the abundance of the various *NGLY1* isoforms. Even though the number of sequenced clones was relatively low and might have missed rare splicing isoforms, larger changes in the abundance would be visible.

**Figure 3:**
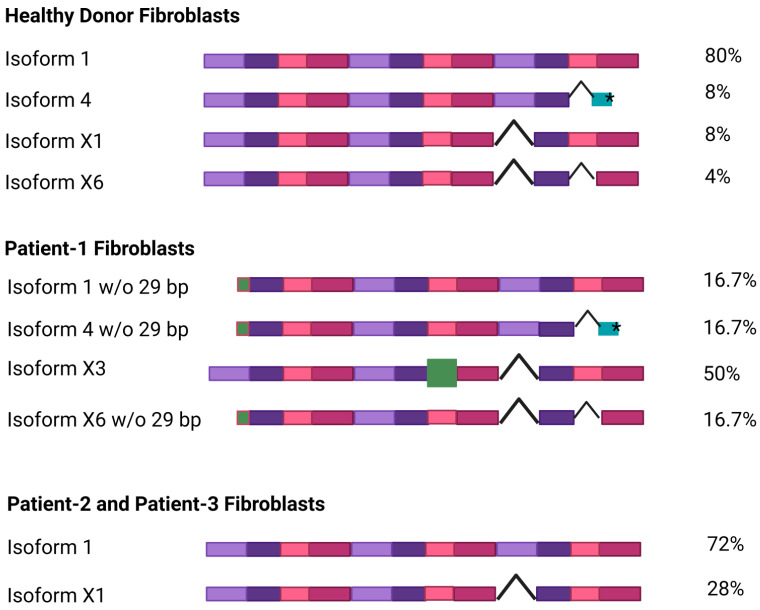
NGLY1 isoforms in healthy donors and NGLY1 patients. Total RNA was isolated and reversely transcribed from dermal fibroblasts, and the coding sequence of *NGLY1* was amplified by PCR. The resulting PCR products were cloned into the pcDNA3 vector and sequenced. Each exon is shown as a colored box; exon skipping is indicated with a hooked line. The green boxes show a truncated exon (isoform 4, *premature stop codon) or an alternative exon (isoform X3). The percentage of each isoform among the sequenced clones is indicated on the right. Figure created with BioRender. Tikkanen, R. (2025) https://BioRender.com/41zizp5.

Isoform 1 was by far the most common transcript in the healthy control cells (80% of the clones, Figure 3). In addition, much lower percentages of isoforms 4, X1, and X6, all of which lack at least one exon, were also detected (8%, 8%, and 4%, respectively; Figure 3). The frameshift variant in exon 9 of patient P1 leads to skipping of exon 9 and thus to increased abundance of isoform X3 (50%), while the deletion at the 5′ end of the other *NGLY1* allele in patient P1 leads to equal percentages of isoforms 1, 4, and X6 (16.7% each). The mRNA expression pattern in the cells of patients P2 and P3 with the homozygous missense mutation was similar to that in healthy cells, with isoform 1 being the most common one (>70%). The Arg390Gln variant in exon 8 therefore does not appear to have a major influence on the alternative splicing of *NGLY1*.

### 3.3. Misprocessing of the Transcription Factor NFE2L1 in Patient Cells and in NGLY1-Knockout HEK293 Cells

**Figure 4:**
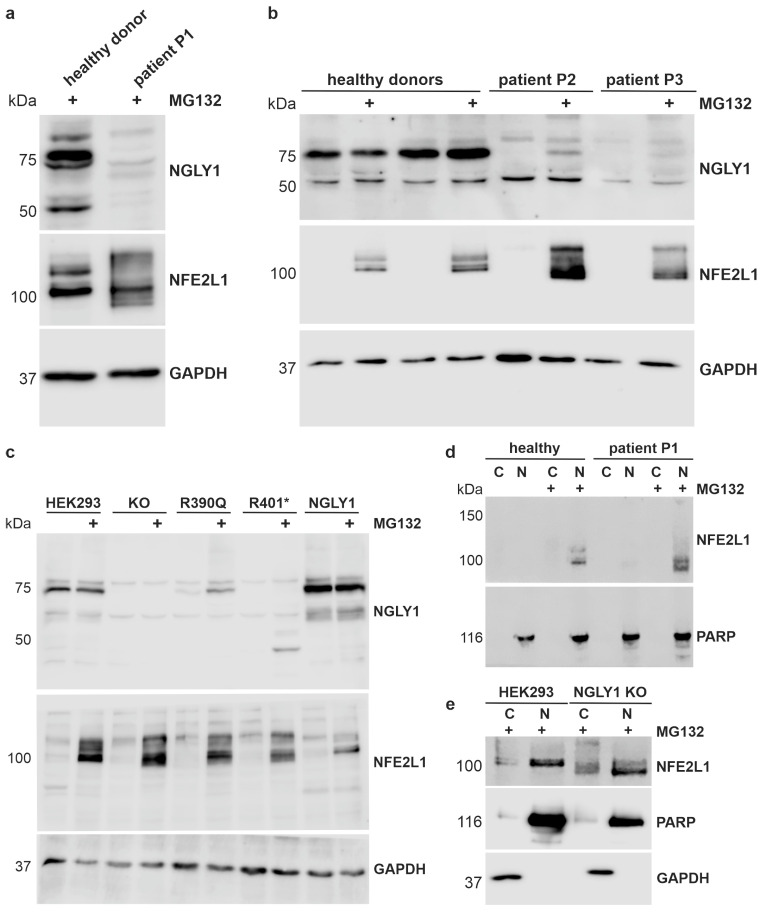
Misprocessing of the transcription factor NFE2L1 in patient cells and in NGLY1-deficient HEK293 cells. (**a,b**) Dermal fibroblasts from healthy donors and NGLY1 patients P1, P2, and P3 were treated overnight with 1 µM MG132. Cell lysates were analyzed by SDS-PAGE and Western blot. GAPDH was used as a loading control. NFE2L1 was only detectable upon proteasome inhibition and showed a misprocessing in NGLY1-deficient cells. (**c**) Stable expression (knock-in) of the WT NGLY1 and the NGLY1 variants R390Q and R401* in NGLY1-deficient HEK293 cells. Parental HEK293 and NGLY1-KO cells were used as controls. After treatment with MG132, NGLY1 and NFE2L1 expression were analyzed by Western blot. The experiments in (**a**–**c**) were performed at least 4 times. (**d,e**) Nuclear extracts were prepared from fibroblasts of patient P1 and a healthy donor (**d**), as well as parental HEK293 and *NGLY1* KO cells (**e**) (three independent experiments each), to study if NFE2L1 is able to reach the nucleus in the absence of NGLY1. Amounts of NGLY1 and NFE2L1 in the nuclear (N) and cytosolic fractions (C) were compared by Western blot. PARP1 was used as a nuclear marker, and GAPDH served as a marker for the cytosolic fraction.

The expression of the NGLY1 protein variants, and the processing and activation of the NGLY1 substrate, NFE2L1, were assessed by Western blot analysis. The degradation of NFE2L1 can be inhibited by the proteasome inhibitor MG132 [34]. A polypeptide corresponding to the NGLY1 isoform 1 was detected in healthy donor fibroblasts at a molecular weight of ca. 75 kDa, and weaker signals were observed at 100 and 50 kDa (Figure 4a). As predicted by the RNA analysis, very little NGLY1 protein was detected in the fibroblasts of patient P1, even after proteasome inhibition (Figure 4a). A protein corresponding to the isoform X3 was not visible, even though it represents about 50% of the NGLY1 mRNA in the patient cells. However, further signals of different sizes were sometimes visible, but these are most likely unspecific signals. In the cells of patients P2 and P3, only minor quantities of the NGLY1 protein were detected, even in the presence of MG132, indicating that the Arg390Gln mutant protein is unstable and may thus undergo an accelerated degradation (Figure 4b).

The transcription factor NFE2L1 needs to be deglycosylated by NGLY1 and proteolytically processed by the protease DDI2 (for a review, see [34]). NFE2L1 exists in different molecular forms, but it can only be detected under conditions where its activity is needed, e.g., upon inhibition of the proteasome. In MG132-treated control cells, the NFE2L1 signal presented as a double band between 100 and 120 kDa, whereas misprocessing of NFE2L1 was found in all three NGLY1 deficiency patients (Figure 4a,b), indicating lack of function of NGLY1. In the patient cells, NFE2L1 signals were also observed at a higher MW than in healthy donors, implicating incomplete deglycosylation [3], and at a lower MW, suggesting misprocessing or abnormal migration on SDS-PAGE gel [7].

As primary patient fibroblasts are not well suited for experiments that require large amounts of material, we generated *NGLY1* knockouts (KOs) in Flp-In™-293 cells by means of CRISPR/Cas9-mediated gene editing. Single-cell clones were isolated, and a clone demonstrating a homozygous insertion of a T after base 515 in exon 4 and lack of NGLY1 expression was chosen for further work (Supplementary Figure S3). The NGLY1 mRNA level is highly reduced (less than 5% of the parental cells, Supplementary Figure S3c), making it unlikely that a truncated protein is expressed from this allele. Based on these *NGLY1* KO cells, we generated knock-in (KI) cells that express the wildtype (WT) NGLY1, the pathogenic missense variant Arg390Gln (found in patients P2 and P3), or the nonsense variant Arg401*, which is the most common pathogenic NGLY1 variant in general. The constructs were stably integrated into the genome of the KO cells using the Flp recombinase, and single-cell clones were chosen. This system provides the advantage of a homogeneously expressing cell population with a moderate overexpression, avoiding the problems associated with transient transfections (inhomogeneous cell population, varying transfection efficiency, etc.). Since the integration takes place at the same chromosomal site, the expression levels are equal between clones, so the protein levels would be principally comparable. The expression of the integrated constructs was tested in MG132-treated cells (Figure 4c). As compared to the WT NGLY1, the expression of the Arg390Gln variant was reduced but detectable, whereas very little NGLY1 protein was detected for Arg401* (Figure 4c). The 45 kDa signal in MG132-treated, R401*-expressing cells most likely represents the truncated NGLY1, but it was not consistently detected in all experiments. The two signals visible in the KO cells are most likely due to antibody background. Consistent with the data from patient cells, expression of the pathogenic variants resulted in NFE2L1 misprocessing and impaired deglycosylation. However, nuclear localization of NFE2L1 was not impaired in the cells of patient P1 (Figure 4d) or in the *NGLY1* KO cells (Figure 4e), despite the lack of NGLY1 expression and function.

### 3.4. Reduced Activity of NFE2L1 and Reduced Expression of NFE2L1 Target Genes in NGLY1-Deficient Cells

**Figure 5:**
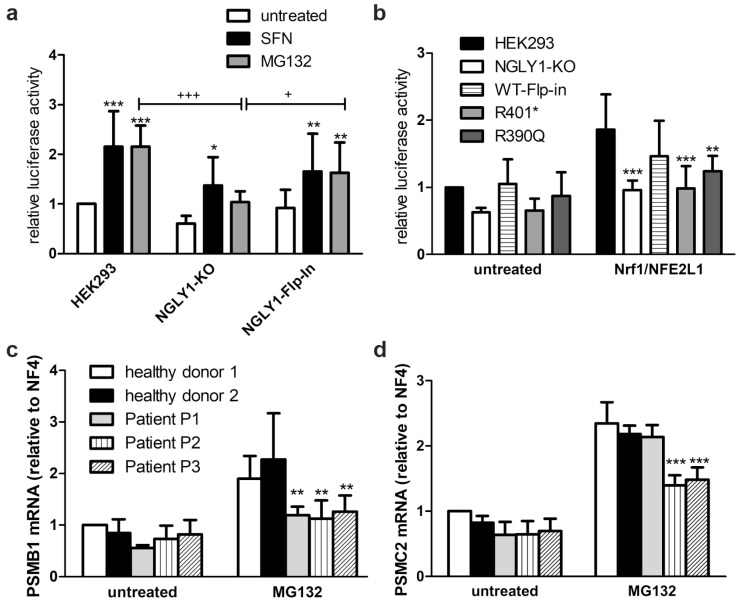
Reduced activity of NFE2L1 and reduced expression of NFE2L1 target genes in NGLY1-deficient cells. (**a**) HEK293, NGLY1-KO, and NGLY1 knock-in cells were transfected with a reporter gene construct containing the antioxidant response element upstream of firefly luciferase. Twenty-four hours after transfection, the cells were exposed to MG132 (1 µM) or sulforaphane (SFN, 10 µM) for 18 h. Firefly luciferase activity was normalized to renilla luciferase. Bars show mean ± SD of 6 independent experiments. Two-way ANOVA against untreated cells. (**b**) HEK293 cells (parental, NGLY1-KO, NGLY1 knock-in, and cells expressing the variants R401* and R390Q) were transfected either with the reporter gene construct in combination with an NFE2L1 expression construct, or the empty vector. Reporter gene activity was measured 48 h after transfection. Bar graphs show means ± SD of 5 independent experiments. Two-way ANOVA against parental cell line. (**c**,**d**) Patient fibroblasts were grown to confluence. RNA was extracted, reverse-transcribed, and amplified by real-time PCR. For normalization, the mean of the reference genes *B2M*, *Rpl13a*, *Ywhaz*, and *GAPDH* was used. Shown are the mean values ± SD of 3 independent experiments. Two-way ANOVA against healthy donors 1 and 2. * *p* < 0.05, ** *p* < 0.01, *** *p* < 0.001 against untreated HEK293; + *p* < 0.05, +++ *p* < 0.001 as indicated.

Misprocessing of NFE2L1 should correlate with its reduced transactivation function as a transcription factor. To study this, the antioxidant response element (ARE), to which both NFE2L1 and the homologous NFE2L2 protein are able to bind, was cloned into the pGL3-promoter upstream of a firefly luciferase, so that an increased luciferase signal should be observed in reporter-transfected cells when NFE2L1 is active. The NFE2L2 activator sulforaphane (SFN) significantly increased the reporter gene activity in both parental and *NGLY1* KO cells. However, proteasomal inhibition by MG132, which results in NFE2L1 activation due to the proteasome bounce-back mechanism [35], only induced a statistically significant increase in the parental cells and in the NGLY1-overexpressing knock-in cells (Figure 5a). *NGLY1* KO cells, and KI cells expressing the pathogenic variants Arg390Gln or Arg401*, already showed a tendency to decreased basal reporter gene activity (Figure 5b), but the reduction was not statistically significant, which may be due to a partial activation of the reporter gene by NFE2L2. However, after overexpression of NFE2L1, which reduces the probability of NFE2L2 binding to the enhancer element, significant differences were observed, and both *NGLY1* KO and KI cells showed significantly reduced reporter gene activity, while the NGLY1 knock-in cells resembled the parental cells (Figure 5b).

The reduced transcriptional activator function of NFE2L1 was confirmed by the reduced mRNA induction of some typical NFE2L1 target genes in the patient fibroblasts, such as the proteasomal subunits PSMB1 (Figure 5c) and PSMC2 (Figure 5d), upon proteasome inhibition by MG132. PSMB1 mRNA levels were significantly reduced in all three patients (Figure 5c), whereas the transcription of PSMC2 was only reduced in patients P2 and P3, but not in P1 (Figure 5d), thus indicating functional differences depending on the underlying genetic variants.

### 3.5. Establishment of an NGLY1 Activity Assay

**Figure 6:**
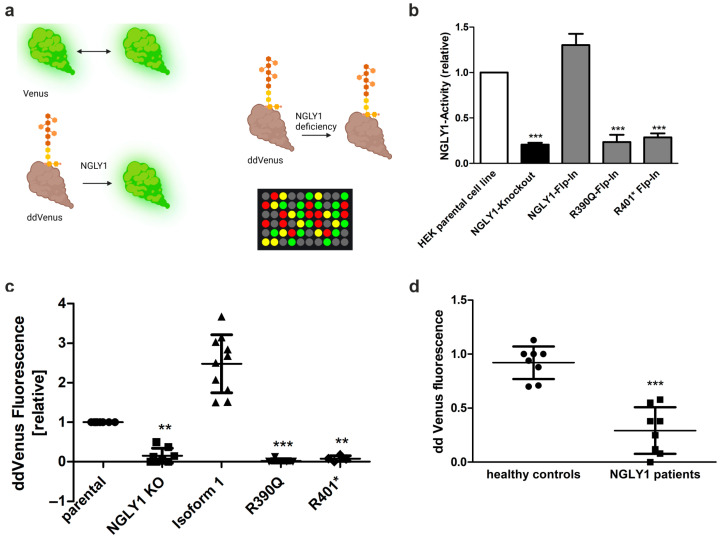
NGLY1 in vivo and in vitro activity assays. (**a**) Assay principle. NGLY1-mediated deglycosylation of the non-fluorescent ddVenus results in the appearance of the fluorescent Venus signal. Fluorescence of unmodified Venus serves as a positive control. (**b**) HEK293 cells were transfected with constructs for either Venus or ddVenus. For normalization, a plasmid encoding mCherry was cotransfected in equal amounts. NGLY1 activity of MG132-treated cells expressing the patient variants Arg390Gln or Arg401* was compared to that of *NGLY1* KO cells. Bars show means ± SD of 3 independent experiments. One-way ANOVA against HEK293 parental cell line (*** *p* < 0.001). (**c**,**d**) NGLY1 in vitro activity assay. Cell homogenates of (**c**) HEK293 cells (n ≥ 4) or (**d**) fibroblasts (n = 8) were incubated with equal amounts of homogenate of cells expressing ddVenus (MG132-treated), and the fluorescence was followed over time. Data show relative mean fluorescence values ± SD of equal amounts of total protein after 48 h of incubation. (**c**) One-way ANOVA against parental cell line; (**d**) unpaired *t*-test against healthy controls. (**c**,**d**) ** *p* < 0.01, *** *p* < 0.001; (**a**) Created by BioRender. Tikkanen, R. (2025) https://BioRender.com/z0q3nku.

NGLY1 activity can be measured by making use of the fact that NGLY1-mediated deglycosylation of an N-glycosylated, engineered variant of the fluorescent protein Venus, called ddVenus, results in the appearance of the fluorescent Venus signal (Figure 6a). Upon deglycosylation, the Asn residue carrying the carbohydrate chain is converted into Asp, which is required for the Venus fluorescence [36]. The endogenous NGLY1 activity in the HEK293 cells was measurable, and the lack of NGLY1 activity in the *NGLY1* KO cells was confirmed by a missing conversion of ddVenus to Venus and very low Venus fluorescence (Figure 6b). The low remaining activity in the KO cells may be due to other enzymes that deglycosylate ddVenus, as described by He et al. [37]. NGLY1 activity was rescued in HEK293 cells with a KI of the NGLY1 isoform 1, but not of the pathogenic variants Arg390Gln or Arg401* (Figure 6b).

Even though the ddVenus-based activity assay is robust and simple, it requires transfection and is thus not suitable for primary cells, such as fibroblasts that are difficult to transfect. We therefore aimed at developing an in vitro test that is also suitable for patient cells and as a diagnostic test. For this purpose, *NGLY1* KO HEK293 cells overexpressing ddVenus were seeded in 10 cm dishes and treated with MG132 overnight to prevent degradation of ddVenus. A cell homogenate was prepared from these cells and used as a substrate for the NGLY1 activity measurement. This homogenate was mixed with homogenates of the target cells whose NGLY1 activity was to be assessed, and the Venus fluorescence was measured over time.

HEK293 cells with endogenous NGLY1 activity were used as a reference (set to 1), and the Venus signals in the other cell lines were normalized to it. Cells with a KI of the WT NGLY1 isoform 1 showed a significant increase (more than 2-fold) in the Venus fluorescence, whereas very low levels of fluorescence were observed in the *NGLY1* KO cells and in the KI cells with the pathogenic NGLY1 variants (Figure 6c). The same assay can also distinguish healthy donors from patient fibroblasts (Figure 6d), even though the total fluorescence values are much lower in primary fibroblasts as compared to the HEK293 cells, which prevents the use of this assay in its current form as a diagnostic test.

### 3.6. In Silico Mutagenesis Reveals the Impact of the R390Q Substitution on NGLY1 Structure

**Figure 7:**
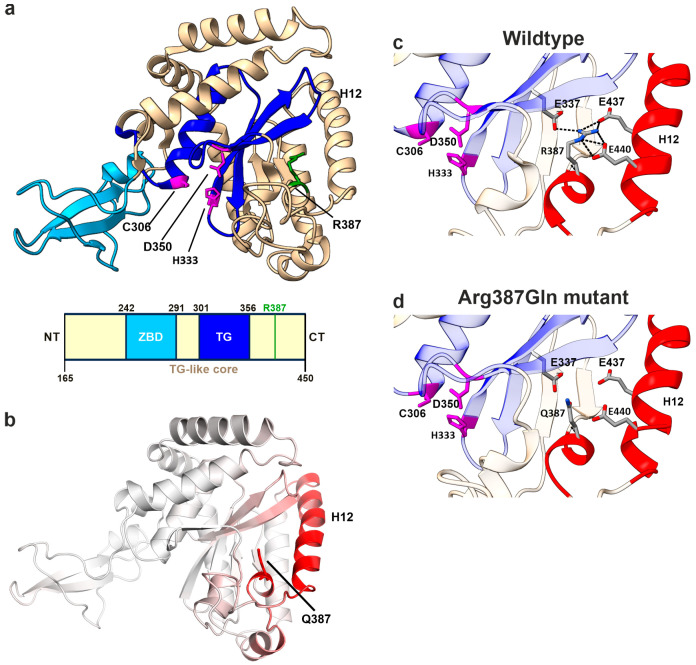
Structural analysis of R387Q variant in mNGLY1. (**a**) Protein structure of mNGLY1 transglutaminase-like-core domain (beige) harboring the catalytic TG domain (blue) and the zinc-binding domain (cyan). The catalytic triad residues Cys306, Asp350, and His333 are indicated in purple and Arg387 in green (PDB: 2F4M). Schematic presentation of the domain structure of mNGLY1 transglutaminase-like-core domain; domains adapted from [4] (TG-like core/ ZBD) and [2] (TG domain); the green bar indicates the position of Arg387Gln (=Arg390Gln in hNGLY1). (**b**) Dynamut analysis of Arg387Gln (n = 3) in mNGLY1 structure reveals changes in the vibrational entropy in the neighboring helix 12 (H12, red), contributing to increased flexibility. The degree of changes in the vibrational entropy correlates with the red color intensity. (**c**) Structural analysis of WT mNGLY1 shows ionic interactions (black dashed lines) between Arg387 and Glu337, and Glu437 and Glu440, stabilizing the protein structure and the adjacent catalytic core. (**d**) The ionic network is lost due to the Arg387Gln exchange. Red color indicates the structural regions with increased flexibility, as predicted by Dynamut.

For a prediction of the impact of the Arg390Gln substitution, in silico mutagenesis was performed. Since no experimentally determined 3-dimensional structure of the human NGLY1 (hNGLY1) protein is available, the mouse NGLY1 (mNGLY1) transglutaminase (TG)-like core domain (PDB ID: 2F4M; residues 165–450 [4]) was used as a structural model. The mouse TG-like core domain comprises two key regions: a zinc-binding domain (ZBD; residues 242–291, [4]) and a TG domain (residues 301–356, [2]). The TG domain includes the catalytic triad with the residues Cys306, Asp350, and His333 in mNGLY1 (Figure 7a).

Amino acid sequence alignment of the TG-like core domain of mNGLY1 with that of hNGLY1 revealed a high similarity, with 94.4% sequence identity (Supplemental Figure S1a). To assess the structural similarity, the amino acid sequence of hNGLY1 (UniProt ID Q96IV0) was used for structure prediction by AlphaFold 3. Overall, the confidence of the prediction is high, except for the disordered region and the linker regions that are more flexible (Supplemental Figures S1c and S2). Importantly, especially for the core of all domains, the confidence is very high. Superimposition of the predicted hNGLY1 structure with the mNGLY1 TG-like core domain showed a strong structural overlap, with a root mean square deviation (RMSD) of 0.944 Å for Cα-atoms (Supplemental Figure S1b). The residue Arg387 in mNGLY1 corresponds to Arg390 in hNGLY1. Based on these findings, we concluded that the experimentally determined mNGLY1 TG-like core structure is a suitable model for studying the structural effects of the human Arg390Gln NGLY1 variant by in silico mutagenesis.

In the subsequent step, the structure of the mNGLY1 TG-like core domain was subjected to in silico mutagenesis with the Dynamut web tool [38] to predict structural and thermodynamic changes associated with the Arg387Gln variant. The analysis revealed an increased molecular flexibility, particularly in the α helix 10 (H10), which contains the mutated residue, and the α helix 12 (H12). The higher flexibility corresponds to an increase in the vibrational entropy (ΔΔSVib) by 0.724 kcal·mol^−1^ × K^−1^, as predicted by the ENCoM method (Figure 7b). Dynamut [27] further predicted that the Arg387Gln exchange exerts a destabilizing thermodynamic effect on the TG-like core, with negative ΔΔG values (WT vs. Arg287Gln) across multiple prediction methods that are included in the Dynamut tool [27]: Dynamut (−1.67 kcal/mol), mCSM (−1.39 kcal/mol), SDM (−1.70 kcal/mol), DUET (−1.572 kcal/mol), ENCoM (−0.579 kcal/mol), and MAESTROweb (−0.61 kcal/mol). These data support our experimental findings showing that the human Arg390Gln variant in hNGLY1 is unstable in cell culture models, including patient fibroblasts.

The structural basis underlying these findings was further investigated by analyzing the intramolecular interactions of Arg387. In the native protein, the positively charged guanidino group of Arg387, located in the helix H10, forms ionic interactions with the negatively charged residues Glu437 and Glu440 in the α helix H12, as well as Glu337 in a β-sheet adjacent to the catalytic center (Figure 7c,d). All three Glu residues are highly conserved across species. These strong ionic interactions are predicted to be essential for stabilizing the position of H12 near the TG domain. In conclusion, Arg387, and its human equivalent Arg390, functions as an anchor that secures the attachment of H12 to the catalytic core, thereby ensuring protein stability and structural integrity.

## 4. Discussion

### 4.1. Urine Glycoasparagines in the Diagnosis of AGU and NGLY1 Deficiency

High levels of glycoasparagines, such as GlcNAc-Asn, in the urine are considered as a hallmark of AGU, a lysosomal storage disorder caused by a deficiency of degradation of N-glycosylated proteins in lysosomes. Patient P1 was also first suggested to suffer from AGU, but her normal AGA activity spoke against this diagnosis, and genetic analysis confirmed the presence of NGLY1 deficiency. Therefore, as both NGLY1-CDDG1 and AGU patients excrete the same compounds in the urine, a thorough differential diagnosis based on, e.g., enzyme activity measurements and genetic testing is recommended to distinguish between these diseases, especially in families with no prior history of either disease.

In addition to GlcNAc-Asn, higher-molecular-weight compounds such as NeuAC-Hex2-HexNAc2-Asn or Neu5Ac1-Hex1-GlcNAc1-Asn can also be detected by liquid chromatography/mass spectrometry [39] or further methods of mass spectrometry [40,41]. Direct comparisons of AGU and NGLY1 patients revealed the same glycoasparagine biomarkers in the urine, but NGLY1-CDDG1 patients appear to excrete significantly less of them [39,42]. However, the amount of glycoasparagine species found in the urine may not be a reliable indication, since AGU patients with milder variants and thus higher residual enzyme activity are likely to exhibit a lower accumulation of glycoasparagines.

### 4.2. NGLY1 Activity Measurement

Beyond direct genetic testing, AGU patients can be reliably identified by AGA enzyme activity measurements in the serum [26], whereas for NGLY1 deficiency, no easy-to-perform routine enzyme activity test is available. The NGLY1 activity assays established to date typically require either transfection of the reporter protein ddVenus [8,23] or the use of a specific cyclic peptide substrate [43,44]. For a detailed comparison of the available methods, we recommend the recent review by Hirayama et al. [45]. The ddVenus assay works well in cell lines that are easy to transfect, but it is not suitable for fibroblasts and other primary cells that are difficult to transfect, so this method cannot be used as a routine diagnostic tool. The methods based on cyclic peptide substrates can measure NGLY1 activity in various materials in a highly sensitive manner, but their use for routine diagnostics is hampered by the fact that the substrate is not commercially available. A highly sensitive enzyme-linked immunosorbent assay for the detection of endogenous NGLY1 activity with very small amounts of sample has been developed, but it also requires the synthesis of an asialoglycopeptide substrate [46].

Our ddVenus-based in vitro assay might offer an alternative method, in which homogenates of a ddVenus-expressing cell line are used to provide the NGLY1 substrate. In order to obtain reproducible results, the reporter cell line should have a consistently high, stable expression of the ddVenus reporter protein, or cell homogenates should be prepared as a larger batch. In addition, a parallel measurement of suitable controls (samples with high or low NGLY1 levels) is recommended. However, it has to be taken into account that all assays with the ddVenus protein require the use of proteasomal inhibitors that are required to stabilize the ddVenus protein. As proteasome inhibition may also stabilize NFE2L1 and the NGLY1 variants, the results may not precisely reflect the specific activity of the NGLY1 variants. Our in vitro assay may be suitable for distinguishing samples with normal/high NGLY1 activity from samples without/with low NGLY1 activity, such as patient samples. However, at present, it is not suitable for measuring the exact NGLY1 residual activities in patients. Future studies should aim at developing an easy-to-use quantitative and sensitive assay that can be used for diagnostic purposes and that would also allow potential treatment success to be followed.

### 4.3. New NGLY1 Variants

We here describe three patients with pathogenic *NGLY1* variants that have not been characterized before. Although these variants are of different natures (i.e., deletion, insertion, or missense variants) and are located at different positions of the *NGLY1* coding sequence, they all lead to a lack of NGLY1 protein expression and misprocessing of the important NGLY1 target, NFE2L1. Our experiments in HEK293 cells show that the novel Arg390Gln variant leads to similar consequences in terms of NGLY1 enzyme activity and NFE2L1 activation to the most common patient variant, Arg401*.

Deglycosylation and proteolytic processing of NFE2L1 can occur independently of each other and are not required to take place in a certain order [7]. Removal of sugars from NFE2L1 by NGLY1 is accompanied by a conversion of Asn to Asp residue, thereby introducing a negative charge that causes a mobility shift of NFE2L1 in SDS-PAGE [7]. This may explain the increased size of NFE2L1 in control cells, which we also observed. Furthermore, we were able to detect NFE2L1 in the nucleus of both healthy and NGLY1-deficient cells. This observation is consistent with the findings of Tachida et al. [7] but contradicts the results of Tomlin et al. [8], who could not find NFE2L1 in the nucleus in the absence of NGLY1. A potential explanation for this discrepancy is the species difference, as Tomlin et al. used mouse cells [8], whereas we and Tachida et al. [7] used human cells.

Our patient P1 is compound heterozygous for a 29 bp deletion and a 1 bp insertion, both of which are expected to prevent NGLY1 expression due to the resulting frameshift. Surprisingly, the 1 bp insertion in exon 9 leads to an altered splicing, although the insertion is not in close proximity to the exon/intron boundaries. The resulting skipping of exon 9 gives rise to high amounts of *NGLY1* isoform X3 at the mRNA level. Although alternative splicing of *NGLY1* and the existence of the X3 isoform have been described previously (reviewed in [5]), we could not detect the X3 isoform at the protein level in patient fibroblasts, even though our NGLY1 antibodies should be able to detect this isoform. Since patient P1 has severe symptoms and exhibits misprocessing of NFE2L1, the X3 protein variant may either be unstable or not functional. Therefore, in terms of potential treatment strategies, this patient is not expected to benefit from approaches that stabilize the NGLY1 protein or aid in assisting its folding (e.g., pharmacological chaperone therapy), but would rather require, e.g., gene therapy or enzyme replacement therapy. In addition, further genetic approaches might be feasible for P1.

In contrast to the insertion variant in P1, the Arg390Gln variant of patients P2 and P3 showed no effect on the splicing of NGLY1 transcripts, and isoform 1 was clearly predominant, as with healthy donor fibroblasts. While the Arg390Gln variant appears not to be enzymatically active, a minor amount of NGLY1 protein was detectable, especially upon treatment of the cells with the proteasome inhibitor MG132. This indicates that the Arg390Gln variant can, in principle, be produced in patient cells, but it is likely to be rapidly degraded by the proteasome. Interestingly, in the ClinVar database, there are entries for genetic variants that result in further amino acid substitutions at position 390, Arg390Pro, Arg390Gly, Arg390Leu, and Arg390Ter, but they have not been studied at the functional level. Thus, the codon for Arg390 may represent a hotspot for *NGLY1* mutations.

### 4.4. Structural Insights into the Molecular Consequences of the Arg390Gln Variant

To better understand the molecular consequences of the Arg390Gln variant on the NGLY1 protein, we performed an in silico mutagenesis analysis of NGLY1 protein structure based on the published structure of the mouse TG-like core domain [4]. The analysis is thus based on a predicted, homologous model and not on experimentally resolved structures. In comparison to the WT mNGLY1 protein, the Arg387Gln protein (equivalent to human Arg390Gln) exhibits an increased flexibility of the helix H12. This is caused by the lack of several ionic interactions of the residue 387/390. In the WT protein, Arg387/390 can interact with three negatively charged glutamate residues, whereas the Gln residue in the mutant protein is not capable of interacting with them due to the missing positive charge in the residue 387/390. It is likely that Arg387, and its human equivalent Arg390, function as anchor residues that secure the positioning of H12 close to the catalytic core, thereby ensuring protein stability and structural integrity. Thus, the pathogenic hNGLY1 variant Arg390Gln is expected to cause an overall loss of stability of the NGLY1 protein.

**Figure 8:**
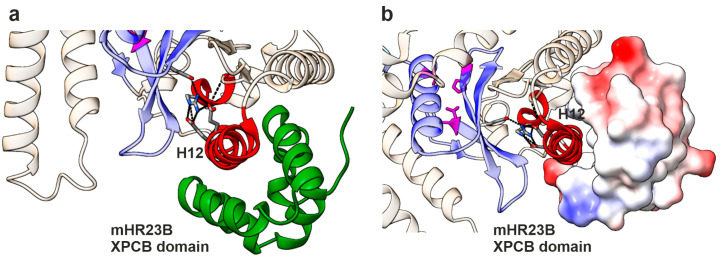
Binding interface of the HR23B-XPCB domain with mNGLY1. (**a**) Protein structure of mNGLY1 transglutaminase-like-core domain in complex with the HR23B-XPCB domain (green); (PDB: 2F4M; [4]). Changes in flexibility due to Arg387Gln mutation predicted by Dynamut are colored in red, including helix 12. The structure reveals H12 as the main interaction site between mNGLY1 and the HR23B-XPCB domain. Color codes as in Figure 7. (**b**) H12 binds in the hydrophobic pocket of the XPCB domain, as shown by electrostatic coloring of the surface mode of HR23B (blue = positive charge; red = negative charge; white = neutral).

Homolog of Rad23 B (HR23B) is involved in the ER-associated degradation (ERAD) pathway, during which it directly interacts with the 26S proteasome and ubiquitinated substrates, mediating proteasomal substrate recognition. However, HR23B is also involved in DNA repair (reviewed in [47]). During nucleotide excision repair (NER), the HR23 xeroderma pigmentosum group C-binding (XPCB) domain binds to the damaged DNA site and starts the global NER. The NGLY1 helix H12 has been shown to be crucial for the binding of NGLY1 to the XPC domain of HR23B [4], and the NGLY1 core domain is structurally very similar to XPC, which is a key component of NER. The helixes H11 and H12 of NGLY1 contain an XPCB association motif, and HR23B mainly interacts with H12 of NGLY1 by hydrophobic interactions (Figure 8a,b). The engagement of Arg390 in the ionic interactions positions the hydrophobic patch of H12 in an optimal orientation for HR23B binding. A disruption of the ionic network surrounding the residue Arg387/390, as caused by its substitution to a Gln residue and loss of the positive charge, leads to increased flexibility of H12 and most likely to an impairment of HR23B binding (Figure 8). This possibility, now based on modeling only, should be experimentally tested in further studies. Thus, based on these structural insights, the Arg387Gln variant may be dysfunctional not only due to its thermodynamic destabilization but also because it loses the ability to interact with HR23B, which is caused by the increased flexibility in H12. This is likely to contribute to the failure of the ERAD pathway in NGLY1 deficiency. However, even though a direct role for NGLY1 in DNA repair/NER has not been shown, it is possible that the HR23B/NGLY1 interaction is required for the regulation of this pathway.

## 5. Conclusions

In this study, we have provided important insights into the consequences of pathogenic NGLY1 variants that cause NGLY1 deficiency. We showed that alterations of *NGLY1* mRNA splicing are caused by genetic variants that do not directly change the splice consensus sites. These findings call for further studies on the abundance of the splice variants in NGLY1 deficiency patient cells. As the function and enzymatic activity of the numerous naturally existing NGLY1 isoforms are not known, further studies should also focus on characterization of these isoforms.

Our elucidation of the structural consequences of the Arg390Gln variant suggested that since this variant is expressed at the protein level, its stabilization, e.g., by chemical or pharmacological chaperones, might be a valid treatment approach for patients such as P2 and P3, and potentially also for patients exhibiting other substitutions of the same amino acid. Drug screenings, both in silico and in vitro, should be performed to identify potential substances that could be used for personalized therapy of patients with Arg390 substitutions. In addition, more general approaches should be envisioned. It has been demonstrated that inhibition or knockout of ENGase, the enzyme that cleaves N-glycans at a more distal site than NGLY1 [48], alleviates some of the disease symptoms in NGLY1 deficiency in mouse models [49], but it is not known if this strategy could be used for treatment of NGLY1 deficiency in humans.

NGLY1 is frequently overexpressed in cancers such as melanoma, and downregulation of NGLY1 has even been suggested as a potential cancer therapy. Knockdown of NGLY1 in melanoma cells caused ER-stress-associated apoptosis and sensitized the cancer cells to DNA alkylating agents, such as dacarbazine or temozolomide [50]. Furthermore, inhibition of NGLY1 by WRR139, a peptide vinyl sulfone, sensitizes leukemia cell lines to proteasome inhibition [8]. Therefore, characterization of NGLY1 functions is not only of interest in NGLY1 deficiency but may have a broader relevance in human diseases.

## Figures and Tables

**Table 1:** Sequences of the primers used in this study.

Primer Name	Sequence 5′–3′
NGLY1-BamHI-UTR-fwd NGLY1-BamHI-P1-fwd NGLY1-XhoI-UTR-rev	CTATAGGATCCGCTGGCGCTCAAGCATGG
	CTATAGGATCCAGTGTGGGACGCGGAGAGCG
	CTATACTCGAGAACTGCCAACTAAGCATGCAC
NGLY1-gRNA-fwd NGLY1-gRNA-rev	CACCGGGACTGAAGAACTTCTAGAA
	AAACTTCTAGAAGTTCTTCAGTCCC
NGLY1-genomic-fwd NGLY1-genomic-rev	AGGCTCTGACACAAATGTGGCT
	TACAAGCCAACGCTTTCTCCTG
NGLY1 c.1169G>A (p.Arg390Gln) fwd NGLY1 c.1169G>A (p.Arg390Gln) rev	GTAGTTGATGTCACTTGGCAATATTCCTGCAAACATGAAG CTTCATGTTTGCAGGAATATTGCCAAGTGACATCAACTAC
NGLY1 c.1201A>T (p.R401*) fwd NGLY1 c.1201A>T (p.R401*) rev	ACATGAAGAGGTGATTGCCTGAAGAACTAAGGTTAAAGAAG CTTCTTTAACCTTAGTTCTTCAGGCAATCACCTCTTCATGT
EcoRI-SS-C-venus-fwd Venus-NotI-stop rev	CTATAGAATTCCCATGGTACCGTGCACGC CTATAGCGGCCGCTTACTTGTACAGCTCGTCCATG
mCherry EcoRI fwd NotI-stop-mCherry-rev	CTATAGAATTCATGGTGAGCAAGGGCGAGGAG CTATAGCGGCCGCTTACTTATAAAGCTCGTCCATGCCG
NFE2L1/Nrf1 HindIII fwd NFE2L1/Nrf1 EcoRI rev	CTATAAAGCTTATGCTTTCTCTGAAGAAATAC CTATAGAATTCTCACTTTCTCCGGTCCTTTG
NGLY1 fwd NGLY1 rev	CAACCTGCAGCCAGTACCCA CAGCAGCAACCGTTGAAGCAG
PSMB1 fwd PSMB1 rev	CCTGCTTGACAACCAGGTTGGT TATGCAGATCCGGAGTGCGTCC
PSMC2 fwd PSMC2 rev	TTGCCCGATCTAGAGGGTCGGA CATACCAGCCTCTGTGCAGACG
B2M fwd B2M rev	AGATGAGTATGCCTGCCGTGTG TGCGGCATCTTCAAACCTCCA
GAPDH fwd GAPDH rev	CATCTTCCAGGAGCGAGATCCC CCAGCCTTCTCCATGGTGGT
RPL13a fwd RPL13a rev	CCTGGAGGAGAAGAGGAAAGAGA TTGAGGACCTCTGTGTATTTGTCAA
YWHAZ fwd YWHAZ rev	AGGTTGCCGCTGGTGATGAC GGCCAGACCCAGTCTGATAGGA

## Data Availability

The original data, excluding direct patient data, are available from the corresponding author upon reasonable request.

## Supplementary Materials

The following supporting information can be downloaded at https://www.mdpi.com/article/10.3390/cells14131036/s1, Figure S1: Comparison of mouse and human NGLY1 structures. Figure S2: Spatial conservation analysis of R390 using a multi-species ortholog comparison. Figure S3: CRISPR/Cas9-mediated knockout of NGLY1 in HEK293 cells. Table S1: Root Mean Square Deviations for the individual NGLY1 domains (mNGLY1 vs. hNGYL1). Table S2: Gene panel for neurodevelopmental disorders.
