# Supplementary material for: Structural and Functional Characterization of N-Glycanase-1 Pathogenic Variants
Source: Cells. 2025 Jul 7;14(13):1036. doi: 10.3390/cells14131036 (PMC12248763; doi:10.3390/cells14131036)
Supplement: Supplementary file 1 [file cells-14-01036-s001.zip › cells-3720275-supplementary.pdf]

Supplementary Figure S1; Banning et al.

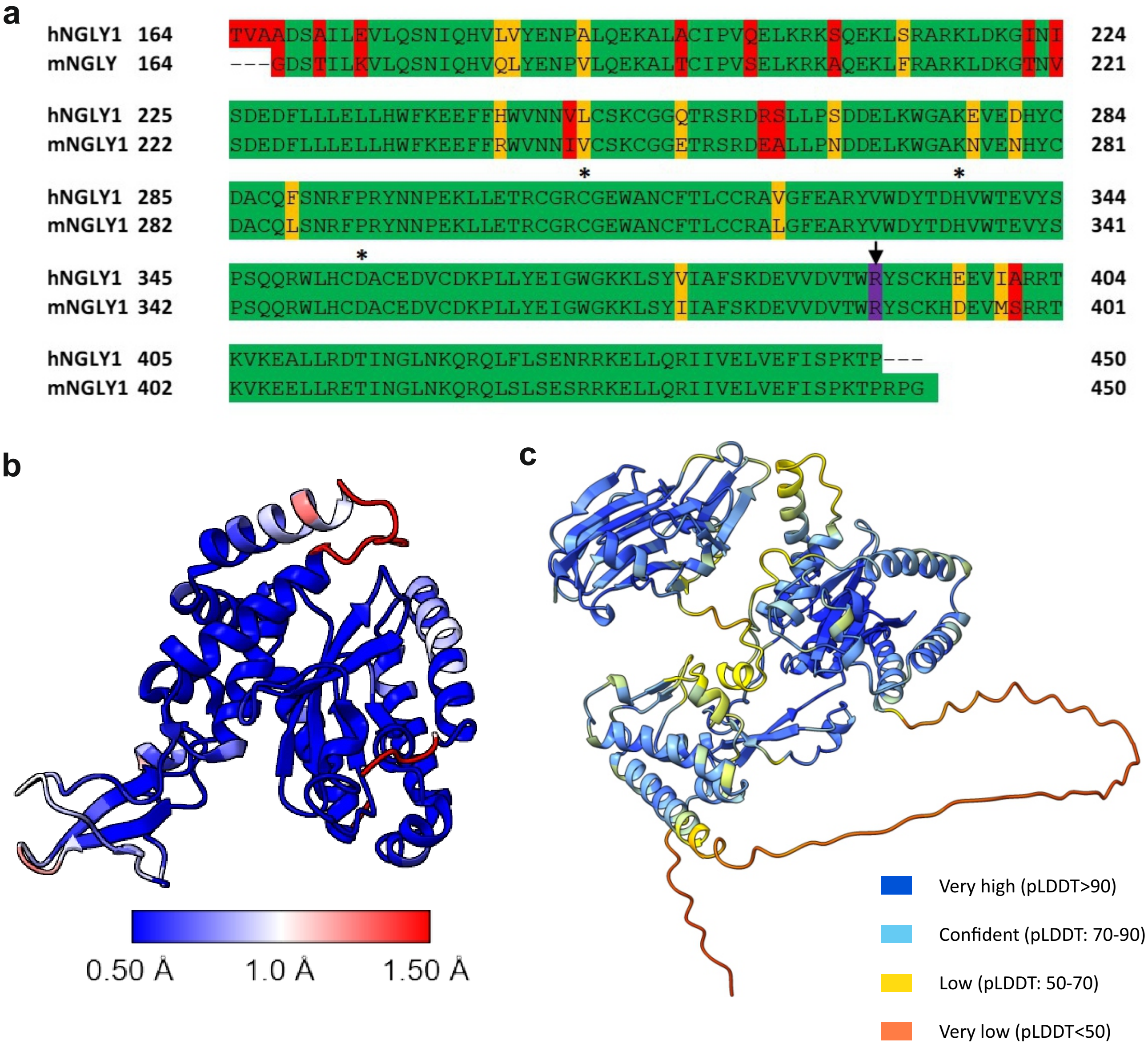

Supplementary Figure S1. Comparison of mouse and human NGLY1 structures. (a) Protein-sequence alignment of the human and mouse NGLY1 transglutaminase-like-cores. * mark the catalytic triad residues; black arrow: position of Arg390/387; green: no amino acid substitution; yellow: conservative substitution; red: non-conservative substitution **(b)** Superimposition of the transglutaminase-like core of hNGLY1 (AF3-prediction) and mNGLY1 (x-ray structure; PDB: 2FM), and calculation of the RMSD of C_{α}-atoms indicates a high structural similarity (RMSD < 0.5 Å = blue; 0.5-1.5 Å = white; >1.5 Å = red). **(c)** Confidence scores of the prediction for the structural elements according to the predicted local distance difference test. Confidence scores are color-coded as indicated.

Supplementary Figure S2; Banning et al.

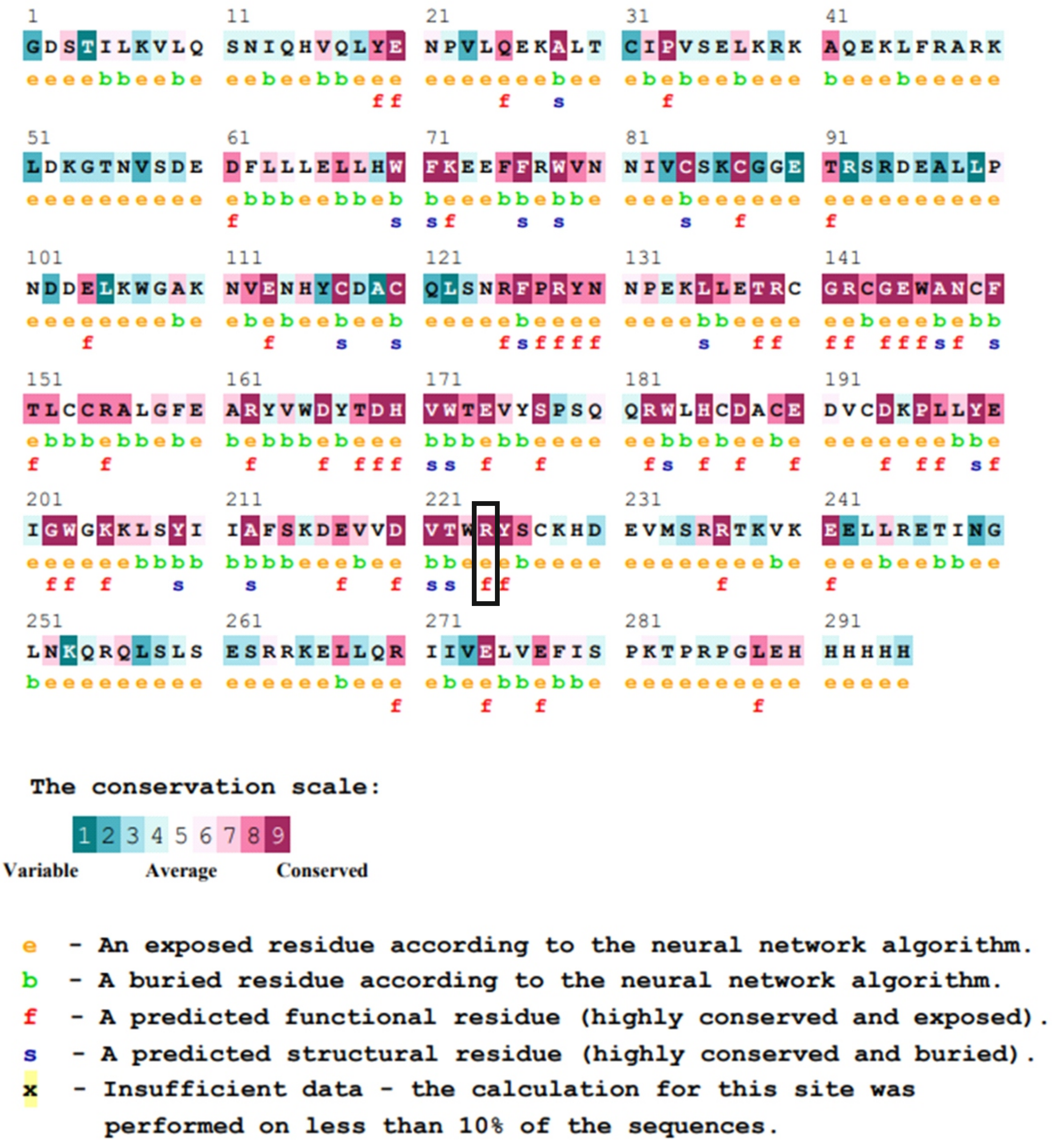

Supplementary Figure S2. Spatial conservation analysis of R390 using a multi-species ortholog comparison. For spatial conservation analysis, the available mouse NGLY1 TG-like domain structure (PDB ID: 2F4M) was analyzed via ConSurf-DB (DOI: 10.1002/pro.3779). The murine sequence was automatically compared to 300 homolog structures from the UniProt database. The R390 amino acid corresponds to position 224 (black box) and is highly conserved (maximum value of 9 out of 9). https://consurf.tau.ac.il/consurf_index.php

Supplementary Figure S3; Banning et al.

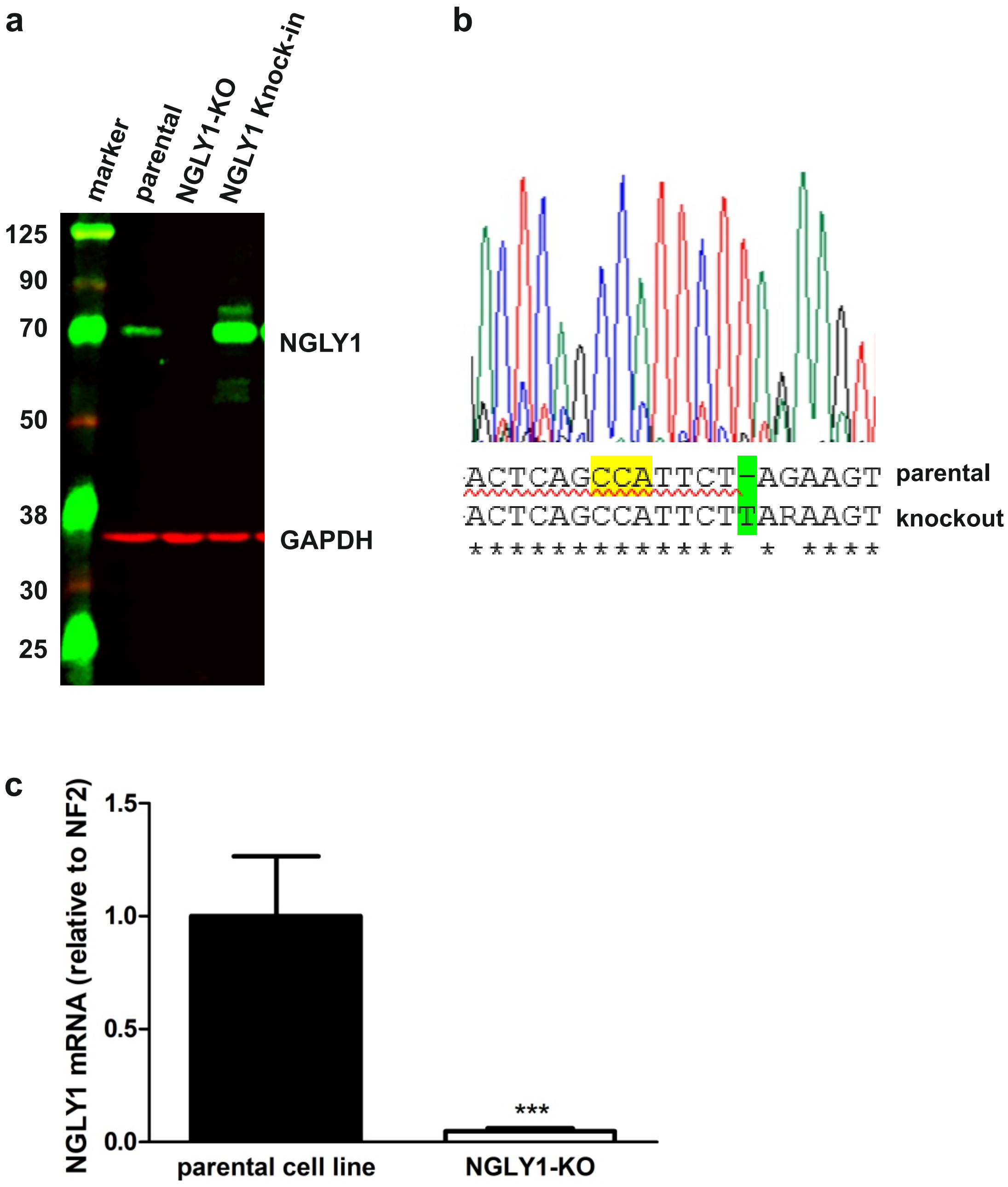

Supplementary Figure S3. CRISPR/Cas9-mediated knockout of NGLY1 in HEK293 cells. (a) Lack of protein expression was confirmed by Western blotting. **(b)** Genetic knockout after insertion of one base (T) was confirmed by sequencing of PCR-amplified genomic DNA surrounding the gRNA targeting site. Knockout cells show a frameshift mutation in the vicinity of the Cas9 PAM sequence (yellow). **(c)** Lack of NGLY1 mRNA expression was confirmed by qPCR with primers targeting exon 3 of NGLY1. NGLY1 mRNA in the knockout cells was reduced to 4.9%. For normalization, the mean of the reference genes B2M and GAPDH was used. Shown are the mean values SD of 5 independent experiments. T-test against parental cell line, ***p<0.001.

Supplementary Table S1: Root Mean Square Deviations for the individual NGLY1 domains (mNGLY1 vs. hNGYL1)

mNGLY1 Domain Name & PDB Number	Sequence Similarity %	RMSD Value
PUB-Domain mNGLY1: PDB 2HPJ	98.9	0.631 Å
TG-like core domain: PDB 2F4M	94.4	0.944 Å
PAW domain: PDB 2G9G	93.8	0.427 Å

Supplementary Table S2: gene panel for neurodevelopmental disorders

AAAS, AARS, AARS2, AASS, ABAT, ABCA2, ABCC8, ABCC9, ABCD1, ABCD4, ABHD12, ABHD16A, ABHD5, ACAD8, ACAD9, ACADM, ACADS, ACAT1, ACBD5, ACER3, ACO2, ACOX1, ACSF3, ACSL4, ACTB, ACTG1, ACTL6B, ACVR1, ACY1, ADA2, ADAM22, ADAMTS10, ADAMTS17, ADAMTS3, ADAR, ADARB1, ADAT3, ADCY5, ADD3, ADGRG1, ADGRV1, ADK, ADNP, ADPRHL2, ADRA2B, ADSL, AFF2, AFF3, AFF4, AFG3L2, AGA, AGO1, AGPAT2, AGRN, AGTPBP1, AHCY, AHDC1, AHI1, AHSG, AIFM1, AIMP1, AIMP2, AK1, AKT1, AKT3, ALDH18A1, ALDH3A2, ALDH4A1, ALDH5A1, ALDH7A1, ALDOA, ALDOB, ALG1, ALG11, ALG12, ALG13, ALG14, ALG2, ALG3, ALG6, ALG8, ALG9, ALKBH8, ALMS1, ALX1, ALX3, ALX4, AMACR, AMER1, AMMECR1, AMPD2, AMT, ANAPC1, ANK3, ANKH, ANKLE2, ANKRD11, ANKRD17, ANO10, ANOS1, ANTXR1, AP1G1, AP1S1, AP1S2, AP2M1, AP3B1, AP3B2, AP3D1, AP4B1, AP4E1, AP4M1, AP4S1, APC2, APTX, ARCN1, ARFGEF1, ARFGEF2, ARG1, ARHGAP31, ARHGDIA, ARHGEF2, ARHGEF6, ARHGEF9, ARID1A, ARID1B, ARID2, ARL13B, ARL3, ARL6, ARMC9, ARNT2, ARSA, ARSE, ARV1, ARX, ASAH1, ASCL1, ASH1L, ASL, ASNS, ASPA, ASPM, ASS1, ASXL1, ASXL2, ASXL3, ATAD1, ATAD3A, ATCAY, ATG5, ATG7, ATIC, ATL1, ATL3, ATM, ATN1, ATP13A2, ATP1A1, ATP1A2, ATP1A3, ATP2A2, ATP6AP2, ATP6V0A2, ATP6V1A, ATP6V1B2, ATP7A, ATP8A2, ATP9A, ATPAF2, ATR, ATRX, AUH, AUTS2, AVIL, AVPR2, B3GALNT2, B3GALT6, B3GAT3, B3GLCT, B4GALT1, B4GALT7, B4GAT1, B9D1, B9D2, BB1Pl, BB5S9, BBS1, BBS10, BBS12, BBS2, BBS4, BBS5, BBS7, BBS9, BCAP31, BCAS3, BCKDHA, BCKDHB, BCKDK, BCL11A, BCL11B, BCOR, BCORL1, BCS1L, BEAN1, BLM, BLOC1S3, BLOC1S6, BMP2, BMP4, BOLA3, BPTF, BRAF, BRAT1, BRF1, BRPF1, BRWD3, BSCL2, BSND, BTD, BUB1B, C12orf4, C12orf57, C12orf65, C19orf12, C2CD3, C5, C8orf37, C9orf72, CA2, CA5A, CA8, CACNA1A, CACNA1B, CACNA1C, CACNA1D, CACNA1E, CACNA1G, CACNA1H, CACNA2D2, CACNB4, CACNG2, CAD, CAMK2A, CAMK2B, CAMK2G, CAMTA1, CANT1, CAPN10, CAPN15, CARS, CARS2, CASK, CASR, CBL, CBS, CC2D1A, CC2D2A, CCBE1, CCDC115, CCDC174, CCDC22, CCDC28B, CCDC32, CCDC47, CCDC78, CCDC88A, CCDC88C, CCM2, CCND2, CCNK, CCTS, CD59, CD96, CDC42, CDC45, CDC6, CDCA7, CDH11, CDH15, CDH2, CDK10, CDK13, CDK19, CDK5, CDK5RAP2, CDK6, CDK8, CDKL5, CDKN1C, CDON, CDT1, CELF2, CENPE, CENPF, CENPJ, CEP104, CEP120, CEP135, CEP152, CEP164, CEP290, CEP41, CEP55, CEP57, CEP63, CEP83, CEP85L, CERS1, CERT1, CFAP43, CHAMP1, CHAT, CHCHD10, CHD1, CHD2, CHD4, CHD7, CHD8, CHKB, CHMP1A, CHMP2B, CHRNA1, CHRNA2, CHRNA4, CHRNB1, CHRNB2, CHRND, CHRNE, CHRNG, CHSY1, CHUK, CIC, CIT, CKAP2L, CLCN2, CLCN3, CLCN4, CLCN6, CLCNKA, CLCNKB, CLDN11, CLDN16, CLDN19, CLIC2, CLN3, CLN5, CLN6, CLN8, CLP1, CLPB, CLPP, CLTC, CNKSR2, CNNM2, CNOT1, CNOT2, CNOT3, CNPY3, CNTN2, CNTNAP1, CNTNAP2, COA7, COA8, COASY, COG1, COG4, COG5, COG6, COG7, COG8, COL13A1, COL18A1, COL3A1, COL4A1, COL4A2, COLEC10, COLEC11, COLGALT1, COLQ, COPB2, COQ2, COQ4, COQ6,COQ8A, COQ8B, COQ9, COX10, COX14, COX15, COX20, COX4I2, COX6B1, COX7B, CP, CPA6, CPE, CPLANE1, CPLX1, CPS1, CPT1A, CPT2, CRADD, CRAT, CRBN, CREBBP, CRIPT, CRLF1, CRPPA, CSF1R, CSNK2A1, CSNK2B, CSPP1, CST3, CSTB, CTBP1, CTC1, CTCF, CTDP1, CTNNA2, CTNNB1, CTNND1, CTSA, CTSD, CTSF, CTU2, CUBN, CUL3, CUL4B, CUX1, CUX2, CWF19L1, CXorf56, CYB5R3, CYFIP2, CYP26B1, CYP27A1, CYP2U1, D2HGDH, DAB1, DACT1, DAG1, DARS2, DBT, DCAF17, DCAF8, DCC, DCHS1, DCPS, DCTN1, DCX, DDB1, DDC, DDHD2, DDOST, DDX11, DDX23, DDX3X, DDX58, DDX59, DDX6, DEAF1, DEGS1, DENND5A, DEPDC5, DES, DGUOK, DHCR24, DHCR7, DHDDS, DHFR, DHH, DHPS, DHTKD1, DHX16, DHX30, DHX37, DIAPH1, DIAPH3, DIP2B, DIS3L2, DKC1, DLAT, DLD, DLG3, DLG4, DLL1, DLL4, DMD, DMPK, DMXL2, DNA2, DNAJC12, DNAJC19, DNAJC21, DNAJC3, DNAJC5, DNAJC6, DNM1, DNM1L, DNMT1, DNMT3A, DNMT3B, DOCK3, DOCK6, DOCK7, DOCK8, DOK7, DOLK, DONSON, DPAGT1, DPF2, DPH1, DPM1, DPM2, DPM3, DPP6, DPYD, DPYS, DPYSL5, DSE, DST, DTNBP1, DVL1, DVL3, DYM, DYNC1/2, DYNC1H1, DYRK1A, EARS2, EBF3, EBP, ECEL1, ECM1, EDC3, EDEM3, EDN3, EDNRB, EED, EEF1A2, EEF2, EFHC1, EFL1, EFNB1, EFTUD2, EGF, EGR2, EHMT1, EIF2AK1, EIF2AK2, EIF2AK3, EIF2B1, EIF2B2, EIF2B3, EIF2B4, EIF2B5, EIF2S3, EIF3F, EIF4A3, EIF4G1, ELAC2, ELOVL1, ELOVL4, ELOVLS, ELP2, EMC1, EMC10, EMG1, EML1, EMX2, ENTPD1, EOGT, EP300, EPB41L1, EPG5, EPHA7, EPM2A, EPRS, ERAL1, ERBB4, ERCC1, ERCC2, ERCC3, ERCC4, ERCC5, ERCC6, ERCC6L2, ERCC8, ERF, ERGIC1, ERLIN2, ESCO2, ETFA, ETFB, ETFDH, ETHE1, EVC, EVC2, EXOC2, EXOC7, EXOSC2, EXOSC3, EXOSC8, EXOSC9, EXT2, EXTL3, EZH2, FA2H, FADD, FAM111A, FAM126A, FAM149B1, FAM20C, FAM50A, FANCA, FANCB, FANCC, FANCD2, FANCE, FAR1, FARS2, FARSB, FASTKD2, FAT2, FAT4, FBXL3, FBXL4, FBXO11, FBXO31, FBXO38, FBXW11, FCSK, FDX2, FGD1, FGD4, FGF12, FGF13, FGF14, FGFR1, FGFR2, FGFR3, FH, FIBP, FIG4, FKBP10, FKRP, FKTN, FLNA, FLNB, FLVCR1, FLVCR2, FMN2, FMR1, FOLR1, FOXG1, FOXP1, FOXP2, FOXP4, FOXRED1, FRAS1, FREM1, FREM2, FRMD4A, FRMPD4, FRRS1L, FTCD, FTL, FTO, FTSJ1, FUCA1, FUS, FUT8, FXYD2, G6PC3, GABBR2, GABRA1, GABRA2, GABRA5, GABRB1, GABRB2, GABRB3, GABRD, GABRG2, GAD1, GAL, GALC, GALE, GALNT2, GALT, GAMT, GAN, GATA6, GATAD2B, GATM, GBA, GBA2, GCDH, GCH1, GCK, GCSH, GDI1, GEMIN4, GEMIN5, GFAP, GFER, GFM1, GJA1, GJB1, GJB3, GJC2, GK, GLB1, GLDC, GLE1, GLI2, GLI3, GLRA1, GLRB, GLS, GLUD1, GLUL, GLYCTK, GM2A, GMNN, GMPPA, GMPPB, GNAI1, GNAO1, GNAQ, GNAS, GNB1, GNB2,

GNB5, GNE, GNPAT, GNPTAB, GNS, GOSR2, GOT2, GPAA1, GPC3, GPHN, GPR88, GPSM2, GPT2, GRIA2, GRIA3, GRIA4, GRID2, GRIK2, GRIN1, GRIN2A, GRIN2B, GRIN2D, GRIP1, GRM1, GRM7, GSS, GSX2, GTF2E2, GTF2H5, GTPBP2, GTPBP3, GUF1, GUSB, HAAO, HACE1, HADH, HADHA, HADHB, HARS, HARS2, HAX1, HCCS, HCFC1, HCN1, HCN2, HDAC6, HDAC8, HECW2, HELLS, HEPACAM, HEPHL1, HERC1, HERC2, HESX1, HEXA, HEXB, HGSNAT, HIBCH, HIKESHI, HINT1, HIST1H1E, HIVEP2, HK1, HLCS, HMGB1, HMGB3, HMGCL, HNMT, HNRNPA1, HNRNPH2, HNRNPK, HNRNPU, HOXA1, HOXA2, HPD, HPDL, HPRT1, HPS1, HPS3, HPS4, HPS5, HPS6, HRAS, HS2ST1, HSD17B10, HSD17B4, HSPA9, HSPB1, HSPB3, HSPB8, HSPD1, HSPG2, HTRA1, HTRA2, HTT, HUWE1, HYLS1, IARS, IARS2, IBA57, ICK, IDH2, IDS, IDUA, IER3IP1, IFIH1, IFT172, IFT27, IFT74, IFT81, IGBP1, IGF1, IGF2, IGHMBP2, IHH, IKBKG, IL11RA, IL1RAPL1, IMPA1, INPP5E, INPP5K, INTS1, INTS8, IQCB1, IQSEC1, IQSEC2, IREB2, IRF2BPL, IRF3, IRX5, ISCA1, ISCA2, ITCH, ITGA7, ITPA, ITPR1, ITSN1, IVD, JAG1, JAM2, JAM3, JARID2, JPH3, KALRN, KANK1, KANSL1, KARS, KAT5, KAT6A, KAT6B, KATNB1, KCNA1, KCNA2, KCNA4, KCNB1, KCNC1, KCNC2, KCNC3, KCNH1, KCNJ1, KCNJ10, KCNJ11, KCNJ2, KCNJ6, KCNK4, KCNK9, KCNMA1, KCNN3, KCNQ2, KCNQ3, KCNQ5, KCNT1, KCNT2, KCTD7, KDM1A, KDM4B, KDM5B, KDM5C, KDM6A, KDM6B, KIAA0556, KIAA0586, KIAA0825, KIAA1109, KIDINS220, KIF11, KIF14, KIF1A, KIF1BP, KIF2A, KIF4A, KIF5A, KIF5C, KIF7, KLC2, KLHL15, KMT2A, KMT2B, KMT2C, KMT2D, KMT5B, KNL1, KPTN, KRAS, KRIT1, L1CAM, L2HGDH, LAGE3, LAMA1, LAMA2, LAMB1, LAMB2, LAMC3, LAMP2, LARGE1, LARP7, LARS, LARS2, LAS1L, LGI1, LGI4, LHX3, LIAS, LIG4, LINGO1, LINS1, LIPT2, LMAN2L, LMBRD1, LMNA, LMNB1, LMNB2, LNPK, LONP1, LRP2, LRP4, LRPPRC, LRRC32, LSS, LYRM7, LYST, LZTFL1, LZTR1, MAB21L1, MAB21L2, MACF1, MADD, MAF, MAGED2, MAGEL2, MAGT1, MAN1B1, MAN2B1, MANBA, MAOA, MAP11, MAP2K1, MAP2K2, MAPK8IP3, MAPKAPK5, MAPRE2, MAPT, MARCHF6, MASP1, MAST1, MAT1A, MATR3, MBD5, MBOAT7, MBTPS2, MCCC1, MCCC2, MCM3AP, MCMS, MCOLN1, MCPH1, MDH2, MDM4, MECP2, MECR, MED12, MED13, MED13L, MED17, MED23, MED25, MED27, MEF2C, MEGF8, MEIS2, METTL23, METTL5, MFF, MFN2, MFSD2A, MFSD8, MGAT2, MGME1, MGP, MICU1, MID1, MID2, MINPP1, MITF, MKKS, MKS1, MLC1, MLPH, MLYCD, MMAA, MMAB, MMACHC, MMADHC, MMUT, MOCS1, MOCS2, MOGS, MORC2, MPDU1, MPDZ, MPLKIP, MPV17, MPZ, MRAS, MRE11, MRM2, MRPL3, MRPS16, MRPS22, MSMO1, MSX2, MTFMT, MTHFR, MTHFS, MTM1, MTO1, MTOR, MTPAP, MTR, MTRR, MTX2, MUSK, MVK, MYBPC1, MYCN, MYH14, MYH3, MYH9, MYMK, MYO18B, MYO5A, MYO9A, MYRF, MYT1L, NAA10, NAA15, NAA20, NACC1, NADK2, NAGA, NAGLU, NAGS, NALCN, NANS, NARS, NARS2, NAT8L, NAXD, NAXE, NBEA, NBN, NCAPD2, NCAPD3, NCAPH, NCDN, NCKAP1, NDE1, NDN, NDP, NDST1, NDUFA1, NDUFA10, NDUFA11, NDUFA12, NDUFA2, NDUFA9, NDUFAF1, NDUFAF2, NDUFAF3, NDUFAF4, NDUFAF5, NDUFAF6, NDUFB3, NDUFB9, NDUFS1, NDUFS2, NDUFS3, NDUFS4, NDUFS6, NDUFS7, NDUFS8, NDUFV1, NDUFV2, NEB, NECAP1, NECTIN1, NEDD4L, NEK9, NEMF, NEU1, NEUROD2, NEXMIF, NF1, NFASC, NFE2L2, NFIX, NFU1, NGF, **NGLY1**, NHEJ1, NHLRC1, NHP2, NHS, NIN, NIPBL, NKAP, NKX2-1, NKX6-2, NLGN3, NLGN4X, NLRP3, NME1, NMNAT1, NONO, NOP10, NOP56, NOTCH3, NOVA2, NPC1, NPC2, NPHP1, NPHP3, NPHP4, NPRL2, NPRL3, NR2F1, NRAS, NRROS, NRXN1, NSD1, NSDHL, NSF, NSMCE2, NSRP1, NSUN2, NT5C2, NTNG2, NTRK1, NTRK2, NUBPL, NUP107, NUP133, NUP37, NUP62, NUP88, NUS1, NXN, OAT, OCLN, OCRL, OFD1, OGT, OPA1, OPHN1, ORC1, ORC4, ORC6, OSGEP, OTC, OTUD5, OTUD6B, OTX2, OXR1, P4HB, P4HTM, PACS1, PACS2, PAFAH1B1, PAGR1, PAH, PAK1, PAK3, PANK2, PARS2, PAX1, PAX2, PAX6, PAX8, PBX1, PC, PCCA, PCCB, PCDH12, PCDH19, PCDHGC4, PCLO, PCNA, PCNT, PDCD10, PDE2A, PDE4D, PDE6D, PDGFB, PDHA1, PDHB, PDHX, PDP1, PDSS1, PDSS2, PDX1, PDXK, PDYN, PEPD, PET100, PEX1, PEX10, PEX11B, PEX12, PEX13, PEX14, PEX16, PEX19, PEX2, PEX26, PEX3, PEX5, PEX6, PEX7, PFN1, PGAP1, PGAP2, PGAP3, PGK1, PGM1, PGM2L1, PGM3, PHACTR1, PHC1, PHF21A, PHF6, PHF8, PHGDH, PHIP,PI4KA, PIBF1, PIDD1, PIEZO2, PIGA, PIGB, PIGC, PIGF, PIGG, PIGH, PIGK, PIGL, PIGN, PIGO, PIGP, PIGQ, PIGS, PIGT, PIGU, PIGV, PIGW, PIGY, PIK3CA, PIK3R2, PIK3R5, PITRM1, PLA2G6, PLAA, PLCB1, PLD1, PLD3, PLEKHG2, PLK4, PLOD2, PLP1, PLPBP, PMM2, PMP22, PMPCA, PMPCB, PNKP, PNP, PNPLA6, PNPO, PNPT1, POC1A, POGZ, POLG, POLG2, POLR1C, POLR1D, POLR2A, POLR3A, POLR3B, POLR3GL, POLR3K, POMGNT1, POMGNT2, POMK, POMT1, POMT2, PORCN, POU1F1, POU4F1, PPIL1, PPOX, PPP1CB, PPP1R15B, PPP1R21, PPP2CA, PPP2R1A, PPP2R2B, PPP2R5D, PPP3CA, PPT1, PQBP1, PRDM12, PRDM8, PREPL, PRICKLE1, PRICKLE2, PRKAR1A, PRKAR1B, PRKCG, PRKDC, PRMT7, PRNP, PRODH, PRPS1, PRR12, PRRT2, PRSS12, PRUNE1, PRX, PSAP, PSAT1, PSMB1, PSMB8, PSMD12, PSPH, PTCH1, PTCHD1, PTDSS1, PTEN, PTF1A, PTPN11, PTPN23, PTRH2, PTS, PUF60, PUM1, PURA, PUS1, PUS3, PUS7, PYCR1, PYCR2, QARS, QDPR, QRICH1, RAB11B, RAB18, RAB23, RAB27A, RAB39B, RAB3GAP1, RAB3GAP2, RAC1, RAC3, RAD21, RAD50, RAF1, RAI1, RALA, RALGAPA1, RANBP2, RAPGEF2, RAPSN, RARB, RARS, RARS2, RBBP8, RBM10, RBM28, RBMX, RBPJ, RCBTB1, RDH11, RECQL4, REEP1, RELN, REPS1, RERE, RETREG1, RFC1, RFT1, RHOA, RHOBTB2, RIN2, RIT1, RLIM, RMND1, RNASEH2A, RNASEH2B, RNASEH2C, RNASET2, RNF113A, RNF125, RNF13, RNF135, RNF168, RNF216, ROGDI, ROR2, RORA, RORB, RPGRIP1L, RPIA, RPL10, RPS19, RPS23, RPS6KA3, RRAS2, RRM2B, RSPRY1, RSRC1, RTEL1, RTN4IP1, RTTN, RUBCN, RUNX2, RUSC2, SACS, SALL1, SALL4, SAMD12, SAMD9, SAMHD1, SARS, SARS2, SASS6,

SATB1, SATB2, SBDS, SBF1, SC5D, SCAPER, SCARB2, SCN11A, SCN1A, SCN1B, SCN2A, SCN3A, SCN4A, SCN8A, SCN9A, SCO1, SCO2, SCP2, SCYL1, SCYL2, SDCCAG8, SDHA, SDHAF1, SDHB, SDHD, SEC24D, SEC31A, SEMA3E, SEMA6B, SEPSECS, SERAC1, SERPINI1, SET, SETBP1, SETD1A, SETD1B, SETD2, SETDS, SETX, SF3B1, SFXN4, SGSH, SH3PXD2B, SH3TC2, SHANK1, SHANK2, SHANK3, SHH, SHMT2, SHOC2, SHROOM4, SIGMAR1, SIK1, SIL1, SIN3A, SIN3B, SIX3, SKI, SKIV2L, SLC12A1, SLC12A5, SLC12A6, SLC13A3, SLC13A5, SLC16A1, SLC16A2, SLC17A5, SLC18A3, SLC19A2, SLC19A3, SLC1A1, SLC1A2, SLC1A3, SLC1A4, SLC20A2, SLC25A1, SLC25A12, SLC25A15, SLC25A19, SLC25A20, SLC25A21, SLC25A22, SLC25A24, SLC25A26, SLC25A3, SLC25A4, SLC25A42, SLC25A46, SLC2A1, SLC30A9, SLC33A1, SLC35A1, SLC35A2, SLC35A3, SLC35C1, SLC39A8, SLC44A1, SLC45A1, SLC46A1, SLC4A4, SLC5A6, SLC5A7, SLC6A1, SLC6A17, SLC6A19, SLC6A3, SLC6A5, SLC6A8, SLC6A9, SLC7A6OS, SLC7A7, SLC9A1, SLC9A6, SLC9A7, SMAD3, SMAD4, SMARCA2, SMARCA4, SMARCB1, SMARCC2, SMARCD1, SMARCE1, SMC1A, SMC3, SMG9, SMOC1, SMPD1, SMPD4, SMS, SNAI2, SNAP25, SNAP29, SNIP1, SNRPB, SNRPN, SNX14, SOBP, SOD1, SON, SOS1, SOS2, SOX10, SOX11, SOX2, SOX3, SOX4, SOX5, SPART, SPAST, SPATA5, SPATA5L1, SPECC1L, SPEN, SPG11, SPG21, SPINK5, SPOP, SPR, SPRED1, SPRED2, SPTAN1, SPTBN1, SPTBN2, SPTBN4, SPTLC2, SQSTM1, SRCAP, SRD5A3, SRP72, SRPX2, SSR4, ST3GAL3, ST3GAL5, STAG1, STAG2, STAMBP, STARD7, STAT2, STIL, STRA6, STRADA, STS, STT3A, STT3B, STUB1, STX11, STX1B, STXBP1, SUCLA2, SUCLG1, SUFU, SUMF1, SUOX, SUPT16H, SURF1, SVBP, SYN1, SYNE1, SYNGAP1, SYNJ1, SYP, SYT14, SYT2, SZT2, TAC3, TACO1, TAF1, TAF13, TAF2, TAF6, TANC2, TANGO2, TARDBP, TARS, TARS2, TAT, TAZ, TBC1D20, TBC1D23, TBC1D24, TBC1D2B, TBC1D7, TBCD, TBCE, TBCK, TBK1, TBL1XR1, TBP, TBR1, TBX1, TCF12, TCF20, TCF4, TCF7L2, TCN2, TCOF1, TCTN1, TCTN2, TCTN3, TDP1, TDP2, TECPR2, TECR, TELO2, TERT, TET2, TFAM, TFAP2A, TFAP2B, TFE3, TFG, TGDS, TGFB1, TGFB2, TGFB3, TGFBR1, TGFBR2, TGIF1, TGM6, TH, THG1L, THOC2, THOC6, THRB, TICAM1, TIMM50, TIMM8A, TINF2, TK2, TKT, TLK2, TLR3, TMCO1, TMEM106B, TMEM107, TMEM13B, TMEM165, TMEM216, TMEM222, TMEM231, TMEM237, TMEM240, TMEM63A, TMEM67, TMEM70, TMEM94, TMLHE, TMTC3, TMX2, TNIK, TNNI2, TNNT3, TNP02, TNRC6A, TNRC6B, TOE1, TOP3A, TOR1A, TP53RK, TP73, TPI1, TPK1, TPM2, TPO, TPP1, TPP2, TPRKB, TRAF3, TRAF3IP1, TRAF7, TRAIP, TRAK1, TRAPPC11, TRAPPC12, TRAPPC2L, TRAPPC4, TRAPPC6B, TRAPPC9, TREM2, TREX1, TRIM32, TRIM71, TRIM8, TRIO, TRIP12, TRIT1, TRMT10A, TRMT1, TRNT1, TRPC3, TRPM6, TRPV4, TRRAP, TSC1, TSC2, TSEN15, TSEN2, TSEN34, TSEN54, TSFM, TSHB, TSPAN7, TTBK2, TTC19, TTC21B, TTC37, TTC5, TTC8, TTI2, TUBA1A, TUBA4A, TUBA8, TUBB, TUBB2A, TUBB2B, TUBB3, TUBB4A, TUBG1, TUBGCP2, TUBGCP4, TUBGCP6, TUFM, TUSC3, TWIST1, TWIST2, TWNK, TYMP, TYROBP, UBA5, UBE2A, UBE3A, UBE3B, UBE4A, UBQLN2, UBR1, UBR7, UBTF, UCHL1, UFC1, UFM1, UFSP2, UGDH, UGP2, UMPS, UNC80, UNC93B1, UPB1, UPF3B, UQCC2, UQCC3, UQCRC2, UQCRQ, UROC1, USP18, USP27X, USP9X, VAC14, VAMP1, VAMP2, VANGL1, VARS, VARS2, VAX1, VIPAS39, VLDLR, VPS11, VPS13A, VPS13B, VPS13D, VPS33B, VPS37A, VPS41, VPS4A, VPS51, VPS53, VRK1, VSX1, VWA3B, WAC, WARS, WARS2, WASF1, WASHC4, WASHC5, WDFY3, WDPCP, WDR11, WDR19, WDR26, WDR35, WDR4, WDR45, WDR45B, WDR62, WDR73, WDR81, WFS1, WIPI2, WLS, WNK1, WNT5A, WWOX, XK, XPA, XPNPEP3, XPR1, XRCC1, XRCC4, XYLT1, XYLT2, YAP1, YEATS2, YIPF5, YME1L1, YWHAG, YY1, YY1AP1, ZBTB11, ZBTB16, ZBTB18, ZBTB20, ZBTB24, ZC3H14, ZC4H2, ZDHHC15, ZDHHC9, ZEB2, ZFYVE26, ZIC1, ZIC2, ZIC3, ZMIZ1, ZMYM2, ZMYND11, ZNF141, ZNF142, ZNF148, ZNF292, ZNF335, ZNF423, ZNF711, ZNHIT3, ZSWIM6
